# Extracellular vesicles of *Clonorchis sinensis* promote the malignant phenotypes of cholangiocarcinoma via NF-κB/EMT axis

**DOI:** 10.1371/journal.pntd.0012545

**Published:** 2024-10-28

**Authors:** Xiaowen Pan, Qing He, Yingxuan Yin, Anyuan Xu, Aoxun Wu, Xueqing Yi, Zifeng Zhong, Yinjuan Wu, Xuerong Li

**Affiliations:** 1 Department of Parasitology, Zhongshan School of Medicine, Sun Yat-sen University, Guangzhou, China; 2 Key Laboratory for Tropical Diseases Control of Ministry of Education, Sun Yat-sen University, Guangzhou, China; 3 Provincial Engineering Technology Research Center for Biological Vector Control, Guangzhou, China; 4 China Atomic Energy Authority Center of Excellence on Nuclear Technology Applications for Insect Control, Beijing, China; 5 Guangzhou Women and Children’s Medical Center, Guangzhou Medical University, Guangzhou, China; 6 Department of Blood Transfusion, The First Affiliated Hospital, Sun Yat-sen University, Guangzhou, China; 7 Department of Basic Medicine, Zhongshan School of Medicine, Sun Yat-Sen University, Guangzhou, China; Instituto de Salud Carlos III, SPAIN

## Abstract

*Clonorchis sinensis* infection is an important risk factor for cholangiocarcinoma (CCA). It has been reported that extracellular vesicles (EVs) are involved in the parasite-host interaction, and EVs of *C*. *sinensis* (*Cs*EVs) can contribute to biliary injuries and inflammation. However, uncertainty surrounds the function of *Cs*EVs in the progression of CCA. In this study, differential ultracentrifugation was used to separate *Cs*EVs from the culture supernatant of *C*. *sinensis* adult worms, and they were then identified by transmission electron microscopy, nanoparticle tracking analysis and proteome assays. CCK8, EdU-488 and colony formation assays were used to explore the effect of *Cs*EVs on the proliferation of CCA cells *in vitro*. Wound healing assays, transwell assays and *in vivo* lung metastasis model were conducted to evaluate the migration and invasion abilities. Moreover, the involvement of EMT process, as well as NF-κB and ERK signaling pathway was assessed. Results showed that *Cs*EVs were successfully isolated and could be taken up by CCA cells, which promoted proliferation by accelerating cell cycle progression. In addition, *Cs*EVs could facilitate cell metastasis by triggering the epithelial-mesenchymal transition (EMT). Mechanistically, activation of NF-κB signaling pathway was involved in the *Cs*EVs-mediated EMT, which could be reversed partly by BAY 11–7082 (an inhibitor of NF-κB). In conclusion, these findings suggested that *Cs*EVs could induce the aberrant proliferation and metastasis of CCA cells by stimulating the NF-κB/EMT axis, providing a novel theoretical explanation for liver fluke-associated CCA.

## Author summary

*C*. *sinensis* infection is closely related to the occurrence of CCA, and the International Agency for Research on Cancer (IARC) has classified *C*. *sinensis* as a class I carcinogen. The exact mechanism of *C*. *sinensis* causing CCA remains to be elucidated, which may be the result of the synergistic effect of multiple factors such as mechanical damage caused by the migration of *C*. *sinensis* adult worms, secondary bacterial infection and inflammatory response, toxicity of the excretory-secretory products, abnormal host immune response and gene mutations. Recently, extracellular vesicles have emerged as a potential mechanism participating in parasite-host interaction, and *Cs*EVs may play an important role in the pathogenesis and carcinogenesis of *C*. *sinensis*. In this study, it was discovered that EVs isolated from the culture supernatant of *C*. *sinensis* adult worms could facilitate the abnormal proliferation of CCA cells by regulating cell cycle progression, and promote the migration, invasion and EMT process through activating the NF-κB signaling pathway. Furthermore, the protein expression profile of *Cs*EVs is revealed by this study, which provides a new perspective for the study of liver fluke-associated CCA and may therefore contribute to the development of novel therapeutic approaches to treat CCA caused by *C*. *sinensis* infection.

## Introduction

*Clonorchis sinensis*, also known as liver fluke, is a neglected food-borne zoonotic parasite that causes clonorchiasis. Eating raw or undercooked freshwater fish or shrimp harboring live metacercariae frequently triggers infection in humans and other mammals [1]. It is speculated that 15 million people are infected around the world, with the majority of cases occurring in China, South Korea, and Vietnam, and more than 200 million individuals are thought to be at risk of infection [1–3]. *C*. *sinensis* adults, which inhabit and lay eggs in the bile duct, can inflame the liver and bile duct system and result in biliary epithelial hyperplasia, periductal fibrosis, hepatic fibrosis, cirrhosis and even cholangiocarcinoma (CCA) [4]. The International Agency for Research on Cancer (IARC) has classified *C*. *sinensis* as a Class I carcinogen due to its close association with CCA [5]. However, the underlying mechanism of CCA induced by *C*. *sinensis* has not been fully elucidated.

Epithelial-mesenchymal transition (EMT) is a crucial process in embryonic development, chronic inflammation, tissue fibrosis and cancer metastasis [6]. At inflammation and wound healing sites, EMT is greatly in favor of reestablishing epithelial and endothelial integrity. However, during the pathological process of tumor progression and metastasis, abnormal reactivation of EMT is related to the malignant properties of tumor cells, including enhancing the invasiveness and migration capability, increasing tumor stemness, and strengthening resistance to chemotherapy and immunotherapy [6–8]. The participation of EMT in the progression of CCA has been proposed to be strongly supported by mounting molecular and cellular evidence [9,10]. However, the relation between EMT and liver fluke-associated CCA is still poorly known and needs further research. It has been demonstrated that excretory-secretory products of *C*. *sinensis* (*Cs*ESPs) facilitate the migration and invasion of CCA cells [11]. Furthermore, our previous study has proved that *C*. *sinensis* granulin (*Cs*GRN), a component of *Cs*ESPs, can promote the metastasis of CCA and hepatocellular carcinoma via EMT process [12–14].

Extracellular vesicles (EVs) are vesicular particles that are released by a wide range of living cells, containing many significant bioactive substances, such as proteins, lipids, and nucleic acids, which are involved in mediating intercellular communication, immunological regulation, as well as other physiological and pathological processes [15]. Increasing evidence points to the possibility that helminths can utilize EVs to facilitate parasite-host interaction and promote their survival and infectivity [16]. Yan et al. [17] discovered that *Cs*EVs had a typical saucer-like structure with a diameter of 30~150 nm, and could promote bile duct injury and the activation of M1-type inflammatory macrophages by delivering microRNA Csi-let-7a-5p targeting at the *Clec7a* and *SOCS1* mediated NF-κB signaling pathway. Another microRNA Csi-miR-96-5p, delivered by *Cs*EVs, can promote tumor proliferation and migration through ferroptosis by regulating the expression of the PTEN/SLC7A11/GPX4 axis [18]. Moreover, *Cs*EVs can promote bile duct epithelial cells to secrete IL-6 and TNF-α by activating Toll-like receptor (TLR) mediated ERK signaling pathway [19]. These results suggest that *Cs*EVs may play a crucial role in the pathogenesis of *C*. *sinensis*, and the proteins and nucleic acids carried by *Cs*EVs may be the key pathogenic molecules. However, the role of *Cs*EVs in promoting the malignant phenotypes of liver fluke-associated CCA remains poorly understood, and the protein composition of *Cs*EVs has not been reported.

In this study, we aimed to elucidate the impact of *Cs*EVs on the malignant progression of CCA and reveal the protein profile of *Cs*EVs to better understand the molecular mechanisms. It was verified that *Cs*EVs could facilitate the aberrant proliferation and metastasis of CCA cells by activating NF-κB and ERK signaling pathway, where EMT played a prominent role. Our findings provide a new perspective for the study of liver fluke-associated CCA and may therefore contribute to the development of novel therapeutic approaches to treat CCA caused by *C*. *sinensis* infection.

## Materials and methods

### Ethics statement

All experimental procedures were approved by the Research Ethics Board of Zhongshan School of Medicine. The animal experiments were approved by the Institutional Animal Care and Use Committee of Sun Yat-Sen University (SYSU-IACUC-2024-000343). All animal studies have followed the ARRIVE guidelines.

### Cell culture and transfection

Human CCA cell lines (RBE and HuCCT1) used in our study were acquired from the Center of Hepato-Pancreato-Biliary Surgery, the First Affiliated Hospital of Sun Yat-sen University. RBE and HuCCT1 cells were cultured in RPMI-1640 medium (Gibco, Carlsbad, USA) with 10% fetal bovine serum (FBS, Gibco, USA) and 1% penicillin-streptomycin (P/S, Hyclone, USA), and maintained at 37°C in an incubator with 5% CO_2_.

Slug-specific small-interfering RNA (siSlug) and scramble negative control of small-interfering RNA (siCon) were designed and synthesized by HanyiBio (Guangzhou, China) (S1 Table). A total of 5×10^5^ *Cs*EVs pretreated RBE and HuCCT1 were transfected using Lipofectamine 3000 (Invitrogen, California, USA) at a final concentration of 100 nM according to the manufacturer’s recommendations. After 48 h of transfection, the cells were collected, and Slug expression was detected by RT-qPCR.

### *C*. *sinensis* and *Cs*EVs preparation

To prepare *Cs*EVs, adult worms of *C*. *sinensis* were extracted from the liver of infective cats purchased from the slaughter house. After being washed five times by sterilized phosphate buffer saline (PBS) containing 5% P/S, the adult worms were transferred to DMEM medium (Gibco, Carlsbad, USA) supplemented with 1% P/S for culture at 37°C with 5% CO_2_ (20~30 worms/2 ml medium in a well). After that, the culture supernatant was collected for *Cs*EVs isolation via differential ultracentrifugation as previously described with some minor adjustments [17,20].

Briefly, the culture supernatant was first centrifuged at 1 500g for 30 min at 4°C, and then the resulting supernatant was centrifuged at 3 500g for another 30 min at 4°C to eliminate worm eggs and excreting waste. Next, the supernatant was gathered and centrifuged at 12 000g for 30 min at 4°C, followed by centrifugation at 20 000g for 1 h at 4°C. Then, the obtained supernatant was filtered through a 0.22 μm PES membrane (Merck Millipore, USA). After that, the supernatant was transferred to the thick wall centrifuge tubes (Beckman Coulter, USA) and then centrifuged at 120 000g for 70 min at 4°C twice in an Optima XE-100 tabletop ultracentrifuge (Swinging bucket rotor, model SW32 Ti, Beckman Coulter, USA). The resultant precipitate containing *Cs*EVs was resuspended in 100 μL PBS and the protein concentration was assessed with a BCA Protein Assay Kit (Thermo Scientific, USA).

### Transmission electron microscopy (TEM)

The morphology of *Cs*EVs was observed by negative-staining TEM. After the ultrafast centrifugation, 20 μL of *Cs*EVs solution was immediately applied to a copper grid and left to rest for 1 min at room temperature (RT). The excess liquid was absorbed by filter paper, and then the grid was negatively stained with 3% phosphotungstic acid aqueous solutions for 1 min and dried at RT. Following, the grid containing *Cs*EVs was imaged using TEM (FEI Tecnai G2 Sprit Twin TEM at 80 kV, USA).

### Nanoparticle tracking analysis (NTA)

The size distribution and concentration of *Cs*EVs were detected by NTA (NanoSight NS300, Malvern Instruments, UK). In brief, *Cs*EVs were diluted 100-fold with ddH_2_O to reach the detection range of the instrument, and then *Cs*EVs diluent was drawn with a 1ml syringe and slowly injected into the sample chamber after it had been cleaned with filtered ddH_2_O. Each sample was measured in triplicate with a 488 nm laser (Blue) and a high-sensitivity sCMOS camera at a camera level of 16 with an acquisition duration of 40 s and a detection threshold setting of 6. The data was analyzed by NTA 3.3 Dev Build 3.3.301 software.

### Proteome assays

The proteome analysis of *Cs*EVs was carried out as previously mentioned [21,22]. In brief, protein sample was extracted from *Cs*EVs using an ultrasonic processor in the presence of lysis buffer containing protease inhibitors, followed by digestion with trypsin (Promega, USA) to obtain tryptic peptides. After desalted on a ZipTip C18 column and vacuum-dried, the peptides were analyzed by liquid chromatography-tandem mass spectrometry (LC-MS/MS) with an EASY nLC system coupled to a Q Exactive HF mass spectrometer (Thermo Scientific, USA). The raw data was searched against a *C*. *sinensis* protein database (uniprot-Proteome ID_UP000286415_20221115.fasta) downloaded from UniProt database using the PEAKS software (Bioinformatics Solutions Inc., Canada). Trypsin was chosen as the enzyme, and at most, 3 missed cleavages per peptide were permitted. Carbamidomethylation was specified as static modifications while oxidation (M) and acetylation (N-term) was set as dynamic modifications. 15 ppm of precursor mass tolerance and 0.03 Da of fragment mass tolerance was used. The peptides were considered when the false discovery rate (FDR) ≤ 0.05.

### *Cs*EVs uptake experiment

To determine whether *Cs*EVs could be incorporated into human CCA cell lines, a PKH26 Red Fluorescent Cell Labeling Kit (Umibio, Shanghai, China) was used to trace *Cs*EVs. In detail, the isolated *Cs*EVs described above were resuspended in 100 μL of PBS, then 200 μL of Diluent C mixed with 1 μL of PKH26 dye were added. The mixture was incubated for 5 min at RT in darkness, and 200 μL of 1% BSA (PBS) was added to terminate staining. The PKH26-labeled *Cs*EVs were washed with PBS by ultracentrifugation at 120 000g for 70 min at 4°C twice to remove excess dye, and resuspended in 200 μL of PBS. PKH26-labeled PBS were used as negative control. Subsequently, the PKH26-labeled PBS or *Cs*EVs were incubated with RBE and HuCCT1 cells previously prepared on coverslips in 6-well plates at 37°C for 12 h. Then, the cells were washed twice with PBS, fixed with 4% paraformaldehyde at RT for 15 min and permeabilized with 0.3% Triton X-100 for 10 min. After the slides dried naturally, add 2 drops of fluorescent mounting medium with DAPI (ZSGB, Beijing, China) to stain nuclei, and the cells were observed using a laser scanning confocal microscope (LSCM, Zeiss, Germany).

### CCK8 assays

Cell Counting Kit 8 (CCK8, Dojindo Laboratories, Kumamoto, Japan) was used to detect cell proliferation. Briefly, RBE and HuCCT1 cells were seeded into 96-well plates at approximately 2×10^3^ cells/well and incubated overnight. Cells were treated with 0 μg/ml, 5 μg/ml, 10 μg/ml and 20 μg/ml *Cs*EVs, respectively. After 0 h, 24 h, 48 h and 72 h of culture, the medium was removed and 100 μL of serum-free RPMI-1640 medium supplemented with 10 μL CCK8 reagent was replaced in each well, and then the cells were incubated in the dark at 37°C for 1 to 4 h. The absorbance value at 450 nm wavelength was detected by a microplate reader (Molecular Devices, CA).

### EdU-488 assays

EdU-488 incorporation assay kit (C0071S; Beyotime, China) was employed to estimate cell proliferation. Briefly, RBE and HuCCT1 cells with or without *Cs*EVs treatment were incubated with EdU medium diluent for 2 h according to the product manual. After being fixed with 4% paraformaldehyde and permeabilized with 0.3% Triton X-100, cells were labeled with EdU-488 through Click reaction, and the cell nuclei were stained with Hoechst 33342. The stained cells were observed under a fluorescence microscope (Leica DMI8, Wetzlar, Germany) and EdU positive cells were analyzed by ImageJ software (National Institutes of Health, USA).

### Colony formation assays

Colony formation assays were used to test the effects of *Cs*EVs on the proliferation ability and tumorigenesis of RBE and HuCCT1 cells. In brief, RBE and HuCCT1 cells with or without *Cs*EVs treatment were seeded into 6-well plates at about 1000 cells/well and cultured for 2 weeks with medium replacement every 3 days. After being washed twice with PBS, cell colonies were fixed with 4% paraformaldehyde and then stained with 1% crystal violet stain solution (Solarbio, Beijing, China) for 30 min. Wash off the excess dye and take photographs. The cell colonies were counted via ImageJ software (NIH).

### Cell cycle analysis

Cell cycle staining kit [MultiSciences (Lianke) Biotech, China] was used to analyze cell cycle distribution. Firstly, 1×10^6^ cells with or without *Cs*EVs treatment were collected and washed once with PBS. Then, Propidium iodide (PI) with RNase A and permeabilization solution were added and incubated at room temperature in darkness for 30 min to stain the cells. The samples were detected using flow cytometry (CytoFLEX S, Beckman Coulter, USA) and the data was analyzed with the ModFit LT 4.1 program (Verity Software House, Topsham, USA).

### Wound healing assays

Wound healing assays were used to analyze the effects of *Cs*EVs on the migration of RBE and HuCCT1 cells. Briefly, RBE and HuCCT1 cells were seeded into 6-well plates at about 5×10^5^ cells/well and incubated to 80% confluence, followed by treatment with or without *Cs*EVs for 24 h. After that, scratches were created vertically with a 200-μL pipette tip and suspension cells were washed off with PBS. The cells were cultured in serum-free RPMI-1640 medium and photographed under a microscope (Leica DMI4000B, Wetzlar, Germany) at 0 h, 24 h and 48 h after scratching. Finally, the scratch area was measured with ImageJ software (NIH).

### Transwell assays

The transwell chambers (Corning, NY, USA) were used to evaluate the migration and invasion ability of RBE and HuCCT1 cells. For the transwell migration assays, 1×10^5^ treated cells were seeded into the upper chamber in serum-free RPMI-1640 medium, while the lower chamber was inserted into 24-well plates supplemented with 600 μL of medium containing 10% FBS. After incubation for 24 h, the migrated cells were fixed with 4% paraformaldehyde for 15 min, and 1% crystal violet solution (Solarbio, Beijing, China) was used to stain. The cells on the upper membrane surface were wiped off, and then the lower membrane surface was randomly imaged with a microscope (Leica DMI4000B, Wetzlar, Germany). The migrated cells were calculated with ImageJ software (NIH). The procedure for the transwell invasion assays was the same as for the migration assays, but the upper chamber was precoated with Matrigel (Corning, NY, USA).

### *In vivo* metastasis assays

Twenty athymic male BALB/c nude mice (5-week-old) were purchased from the Laboratory Animal Center of Sun Yat-sen University. The following experimental protocol was reviewed and approved by the Institutional Animal Care and Use Committee (IACUC), Sun Yat-sen University (No. SYSU-IACUC-2024-000343). For the lung metastasis model, approximately 2 × 10^6^ control or *Cs*EVs treated-RBE cells were resuspended in 200 μL PBS and injected into the nude mice through the tail vein (n = 10/per group). After about 4 weeks of inoculation, the mice were sacrificed by cervical dislocation after anesthetization via intraperitoneal injection with 1% sodium pentobarbital, and their lungs were separated for the subsequent histopathologic analyses with hematoxylin-eosin staining.

### RT-qPCR

Total RNA was extracted from cells using the EZ-press RNA Purification Kit (EZBioscience, USA) according to the manufacturer’s instructions, and an Epoch Microplate Spectrophotometer (BioTek, USA) was used to evaluate the RNA quality by determining parameters of concentration and purity. Complementary DNA (cDNA) was synthesized using 5× *Evo M-MLV* RT Master Mix (AGBio, China), and quantitative analysis was performed using SYBR Green Premix Pro Taq HS qPCR Kit (AGBio, China) on a CFX96 Real-Time PCR system (Bio-Rad). The specific primers used in this study were synthesized by Sangon Biotech (Shanghai, China) and shown in S2 Table. Relative mRNA expression was calculated using the 2^-ΔΔCt^ formula as in a previous study [12]. All experiments were performed in triplicate.

### Western blot

Western blot was carried out in accordance with a previous study [13]. Briefly, total protein was extracted from cells using radioimmunoprecipitation assay (RIPA) lysis buffer (Beyotime, Shanghai, China) containing protease inhibitors and phosphatase inhibitors. Protein samples were quantified with a BCA Protein Assay kit (Thermo Fisher Scientific, USA) and denatured with the 5×SDS loading buffer at 100°C for 10 min. A total of 30 μg protein from each sample was separated by SDS-PAGE and transferred onto polyvinylidene difluoride (PVDF) membranes (Millipore, Bedford, USA). After blocking with 5% skim milk for 2 h, the membranes were incubated with the corresponding primary antibodies (S3 Table) at 4°C overnight, including anti-E-cadherin (1:2,000), anti-N-cadherin (1:1,000), anti-vimentin (1:1,000), anti-slug (1:1,000), anti-CDK2 (1:1,000), anti-CDK6 (1:2,000), anti-CyclinD1 (1:1,000), anti-CyclinD3 (1:2,000), anti-p-ERK (1:2,000), anti-ERK (1:1,000), anti-p-IKKα/β (1:1,000), anti-IKKα (1:1,000), anti-IKKβ (1:1,000), anti-p-IKBα (1:1,000), anti-IKBα (1:1,000), anti-p-p65 (1:1,000), anti-p65 (1:1,000), and anti-GAPDH (1:1,000). Subsequently, the membranes were supplemented with horseradish peroxidase (HRP)-conjugated secondary antibodies (1:5,000; Proteintech, Wuhan, China) for 2 h incubation at RT. The enhanced chemiluminescence reagent (ECL; EpiZyme, Shanghai, China) was used for development and ImageJ software (NIH) was used for quantification.

### Statistical analysis

Statistical analysis was performed by GraphPad Prism 8.0 (GraphPad, San Diego, USA). In the case of comparison of two groups, the differences were evaluated using a two-tailed Student’s *t*-test, and One-way ANOVA analysis was used for comparison of differences among more than two groups. All results were from three independent experiments and the data were expressed as Mean ± SD. The differences were statistically significant under the following circumstances: *P < 0.05, **P < 0.01, ***P< 0.001, ****P<0.0001.

## Results

### Purification and identification of *Cs*EVs

**Fig 1:**
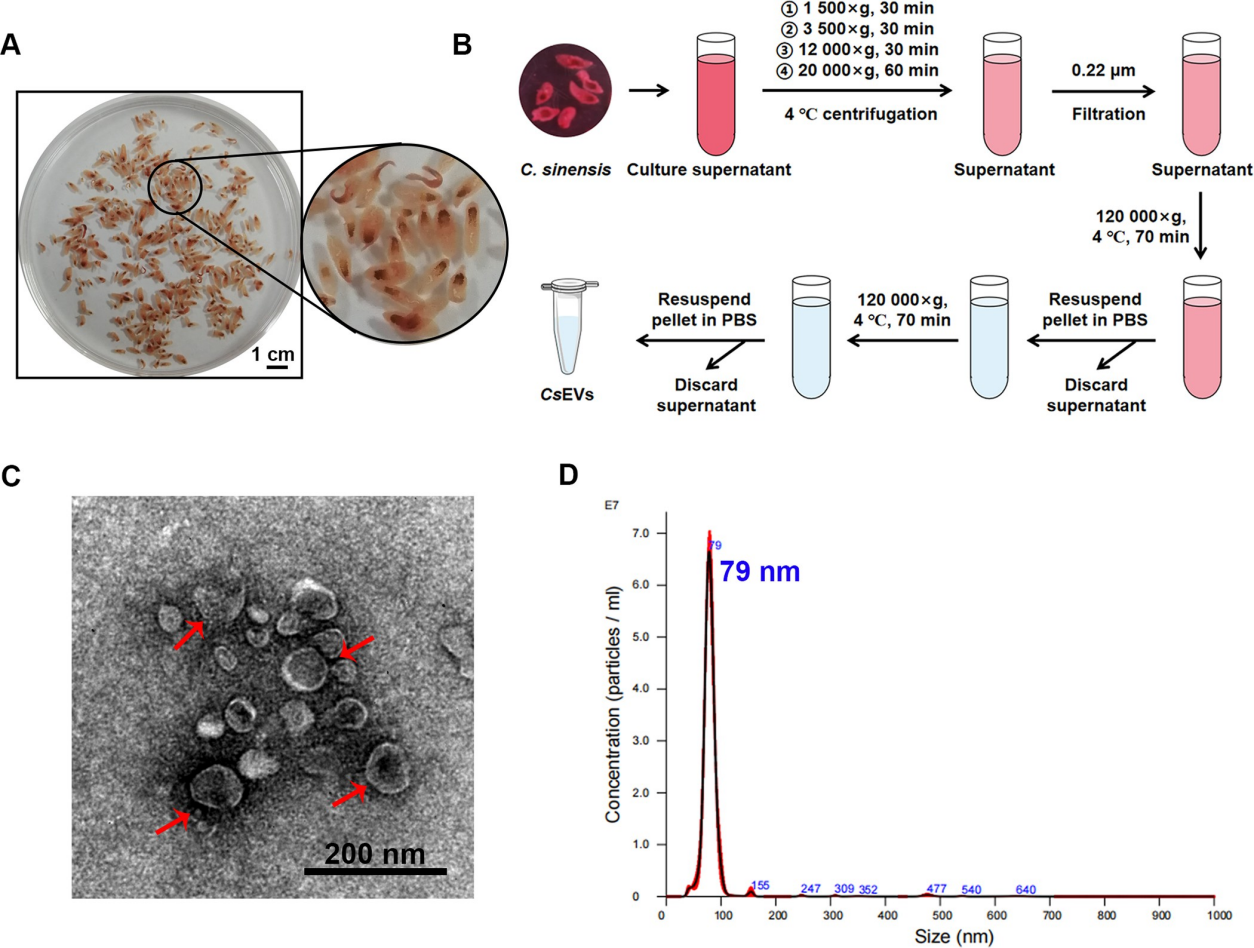
Purification and identification of EVs derived from *C*. *sinensis*. **(A)** *Cs*EVs isolated from the liver of infected cats. Scale bar: 1 cm. **(B)** Procedure for purification of *Cs*EVs using differential ultracentrifugation. **(C)** Morphological identification of *Cs*EVs by negative-staining TEM, and typical vesicular structures can be observed. Scale bar: 200 nm. **(D)** Nanoparticle tracking analysis of purified *Cs*EVs. The concentration and size distribution were plotted with peaks labeled with nm size.

To obtain *Cs*EVs, we first collected the adult worms of *C*. *sinensis* from the liver bile duct of the infected cats, and then cultured them in DMEM medium after washing procedures (Fig 1A). Next, *Cs*EVs were separated from the culture supernatant via differential ultracentrifugation (Fig 1B). Negative-staining TEM was used to determine the morphological properties of *Cs*EVs, which showed the typical cup-shaped or rounded structures of extracellular vesicles with a double-layer membrane and the diameter was between 30~150 nm (Fig 1C). Furthermore, the size distribution detected by NTA revealed a consistent result with a peak at 67.1 to 92.5 nm in diameter, and the concentration of *Cs*EVs in our preparation was 70.6 particles per frame, which was equal to a concentration of 1.5×10^11^ particles/mL before 100-fold dilution with ddH_2_O (Fig 1D). These results indicated that *Cs*EVs were successfully isolated from the supernatant.

### Proteomic analysis of *Cs*EVs

To elucidate the protein composition of isolated *Cs*EVs, LC-MS/MS assays were performed. A total of 114 proteins present within the *Cs*EVs fraction were identified, of which 11 were uncharacterized proteins (S4 Table). Among the characterized 103 proteins, some of the most frequently identified proteins in EVs, such as tetraspanin, annexin, tubulin, actin and heat shock protein (HSP) 90, which were implicated in EVs biogenesis, secretion and cell targeting, were also found to exist in *Cs*EVs [23]. Additionally, some proteins, such as myoferlin, leucine aminopeptidase 2 and cathepsin B, matched with those previously identified in *Cs*ESPs, and may be related to the pathogenesis and oncogenicity of *C*. *sinensis* [22].

**Fig 2:**
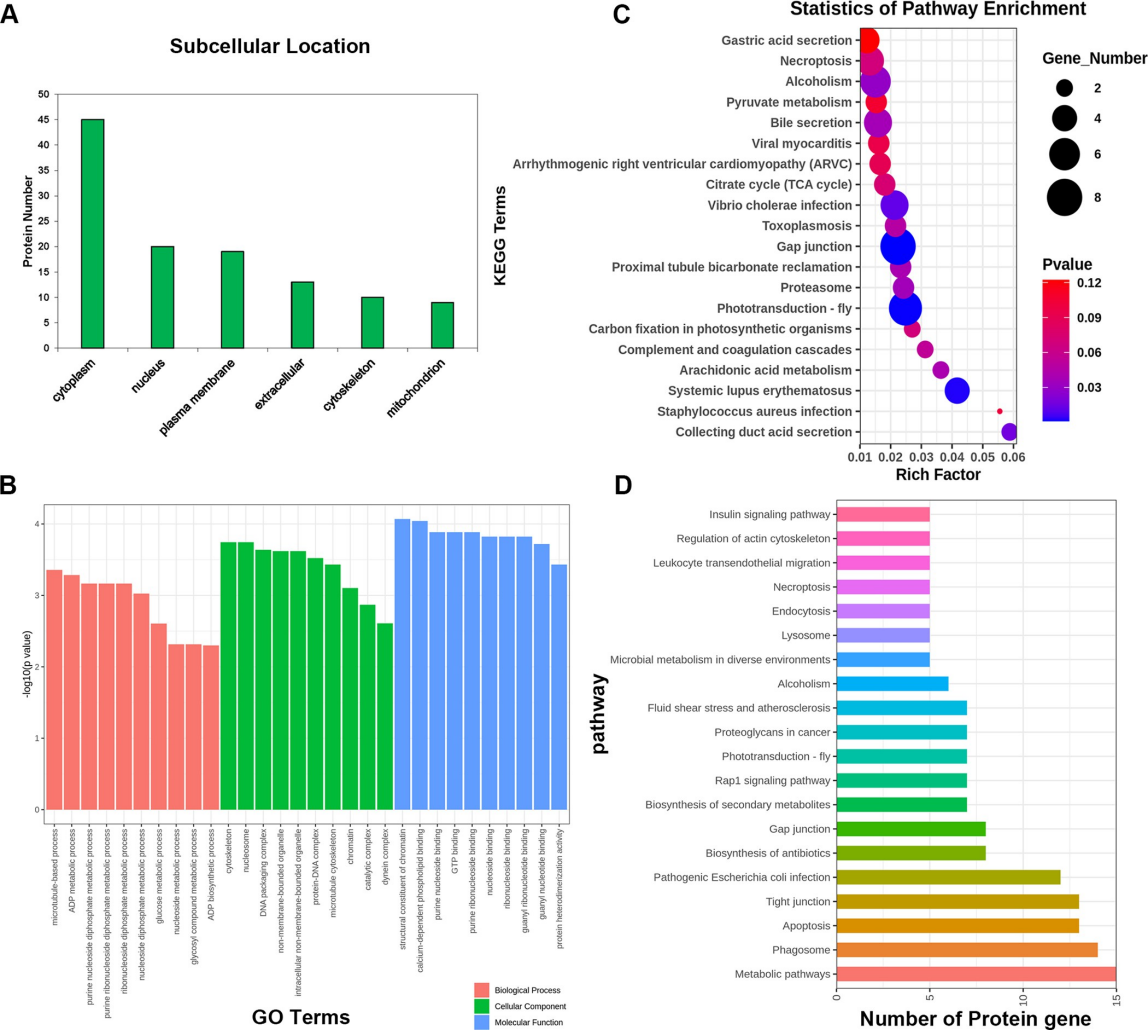
Bioinformatics analysis of the proteins contained in *Cs*EVs. **(A)** Subcellular location of the *Cs*EVs proteins. Bar graph representing the abundance of *Cs*EVs proteins at different subcellular locations. **(B)** Gene ontology (GO) annotation of the *Cs*EVs proteins. Enrichment analyses by biological process, cell component, and molecular function, respectively. **(C)** Bubble map of KEGG pathway of the *Cs*EVs proteins (Top 20). X-axis indicates the rich factor; Y-axis indicates the terms of KEGG pathway. The dot size means the protein number and dot color indicates the p-value. **(D)** Distribution of the enriched KEGG pathways (Top 20). Bar chart showing the abundance of *Cs*EVs proteins at different pathways.

Subcellular localization of the *Cs*EVs proteins was predicted using WoLF PSORT software [24]. The majority of the proteins were localized in the cytoplasm (45), with the remaining proteins sequentially deriving from the nucleus (20), plasma membrane (19), extracellular space (13), cytoskeleton (10), and mitochondrion (9) (Fig 2A). Then, the identified *Cs*EVs proteins were annotated using the UniProt-GOA database (http://www.ebi.ac.uk/GOA/) and classified based on three categories. In the case of biological processes, *Cs*EVs proteins were primarily enriched in the metabolic processes. GO enrichment of cellular components revealed that most of the *Cs*EVs proteins were found to be cytoskeletal and nucleosome proteins. When it comes to molecular function, *Cs*EVs proteins were considerably enriched in binding functions (Fig 2B). The KEGG analysis showed that *Cs*EVs proteins were mainly abundant in gap junction, collecting duct acid secretion, bile secretion, arachidonic acid metabolism, etc., and most were predicted to be associated with the metabolic pathways (Fig 2C and 2D).

### *Cs*EVs can be internalized by CCA cell lines

**Fig 3:**
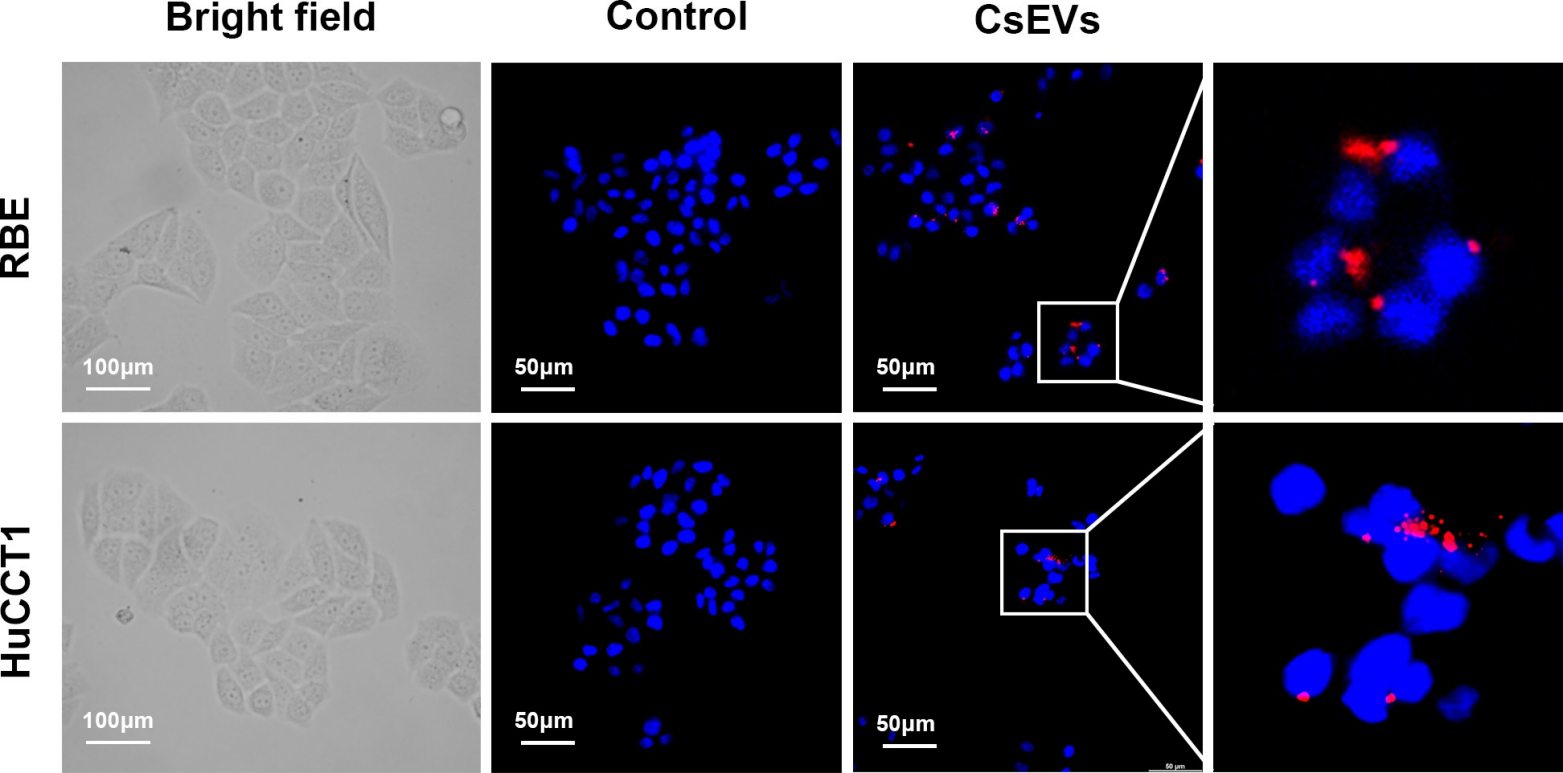
*Cs*EVs can be internalized by CCA cells. *Cs*EVs were stained with PKH26 and then incubated with RBE and HuCCT1 cells for 12 h. Laser scanning confocal microscope was used to detect the *Cs*EVs internalization. Bright field showed the size and ratio of nuclei to cytoplasm in RBE and HuCCT1 cells. Scale bar: 100 μm. Red fluorescence representative PKH26-labeled *Cs*EVs, and the blue are cell nuclei. As shown in the images, *Cs*EVs were localized in the cytoplasm surrounding the nucleus. Scale bar: 50 μm.

To clarify the effects of *Cs*EVs on the biological function of CCA cells, we first examined whether *Cs*EVs could be captured by RBE and HuCCT1 cells. PKH26, a lipophilic red fluorescent dye, was used to label *Cs*EVs specifically. Then these PKH26-labeled *Cs*EVs were incubated with RBE and HuCCT1 cells for 12 h. PKH26-labeled PBS treated cells were used as negative control. As shown in Fig 3, red fluorescence signals were clearly observed in RBE and HuCCT1 cells around the nucleus (blue) by using a laser scanning confocal microscope (LSCM), suggesting that *Cs*EVs can be internally absorbed by CCA cells.

### *Cs*EVs induce the proliferation of CCA cells *in vitro*

**Fig 4:**
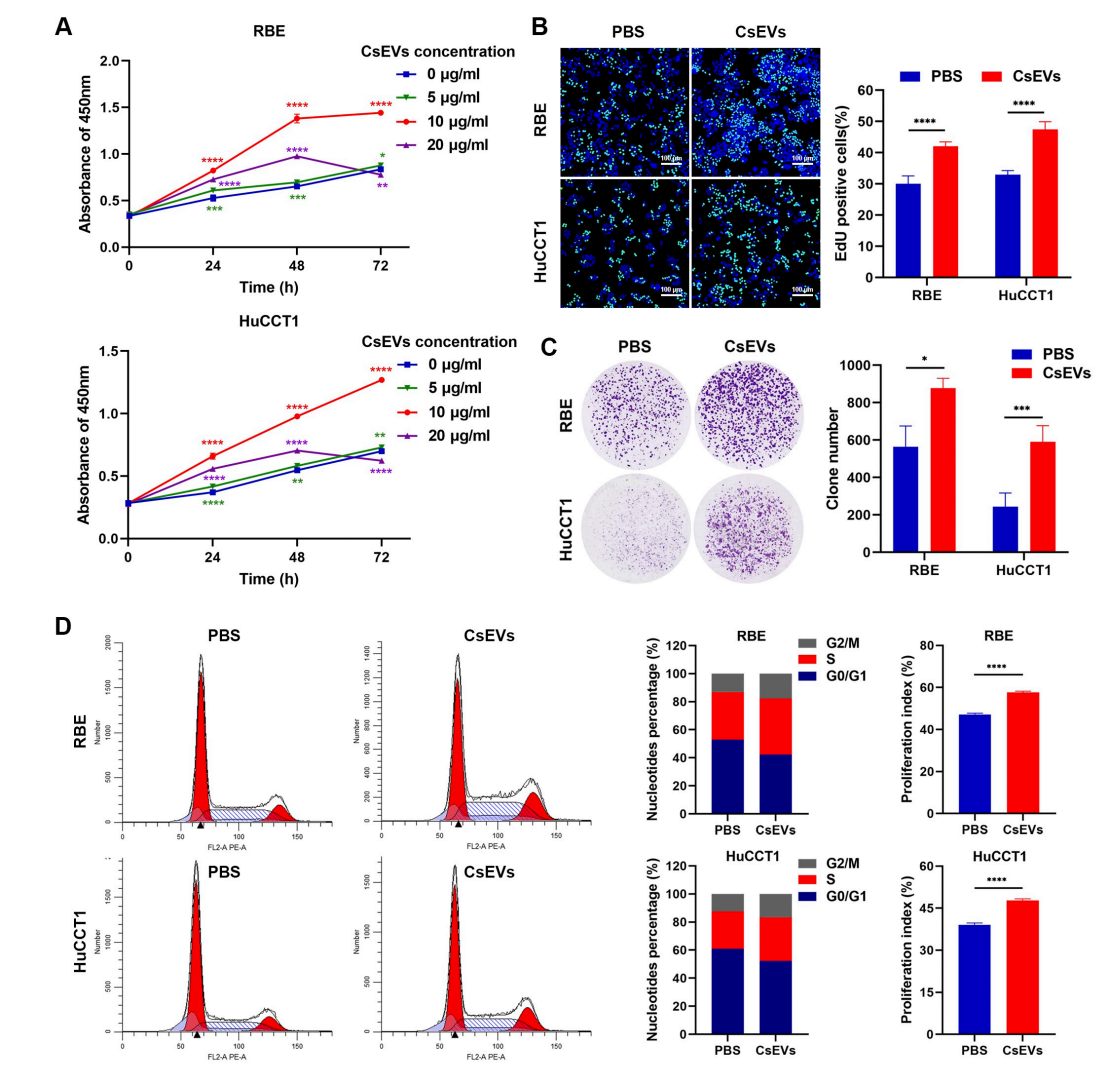
*Cs*EVs induce the malignant proliferation of CCA cells. **(A)** CCK8 assay was employed to detect cell proliferation. RBE and HuCCT1 cells were treated with different concentrations of *Cs*EVs, respectively, and detected at 0, 24, 48 and 72 h after treatment. **(B)** EdU-488 assay and **(C)** colony formation assay were conducted to determine the proliferation capacity of RBE and HuCCT1 cells after treatment with or without 10 μg/ml of *Cs*EVs for 48 h, respectively. Representative images were shown and the summary results were from three independent experiments. Scale bar: 100 μm. **(D)** Cell cycle distribution was analyzed after treatment with or without 10 μg/ml of *Cs*EVs for 48 h by flow cytometry. The percentage of cell cycle phase and the proliferation index (PI) were calculated and shown. PI = (S+G2/M)/(G0/G1+S+G2/M).

Next, we explored whether *Cs*EVs could affect the growth of CCA cells. As demonstrated by CCK8 assays, the proliferation ability of RBE and HuCCT1 cells treated with 10 μg/mL of *Cs*EVs was significantly improved compared with other groups (*p* < 0.0001), while the effect was lost at 5 μg/mL and substantially dampened at 20 μg/mL. (Fig 4A). Similarly, *Cs*EVs treatment increased the colony formation numbers and resulted in more EdU positive cells relative to the control (Fig 4B and 4C), indicating that *Cs*EVs facilitated the proliferation of CCA cells. Moreover, to determine how the proliferation of CCA cells was induced by *Cs*EVs, we further conducted the cell cycle analysis via flow cytometry, which showed that there was elevated cell aggregation in both S and G2/M phase with an increased proliferation index after *Cs*EVs treatment (Fig 4D). These results demonstrate that *Cs*EVs can promote cell division and proliferation by accelerating cell cycle progression.

### *Cs*EVs facilitate the proliferation and oncogenicity of CCA cells by regulating cell cycle-related molecules

**Fig 5:**
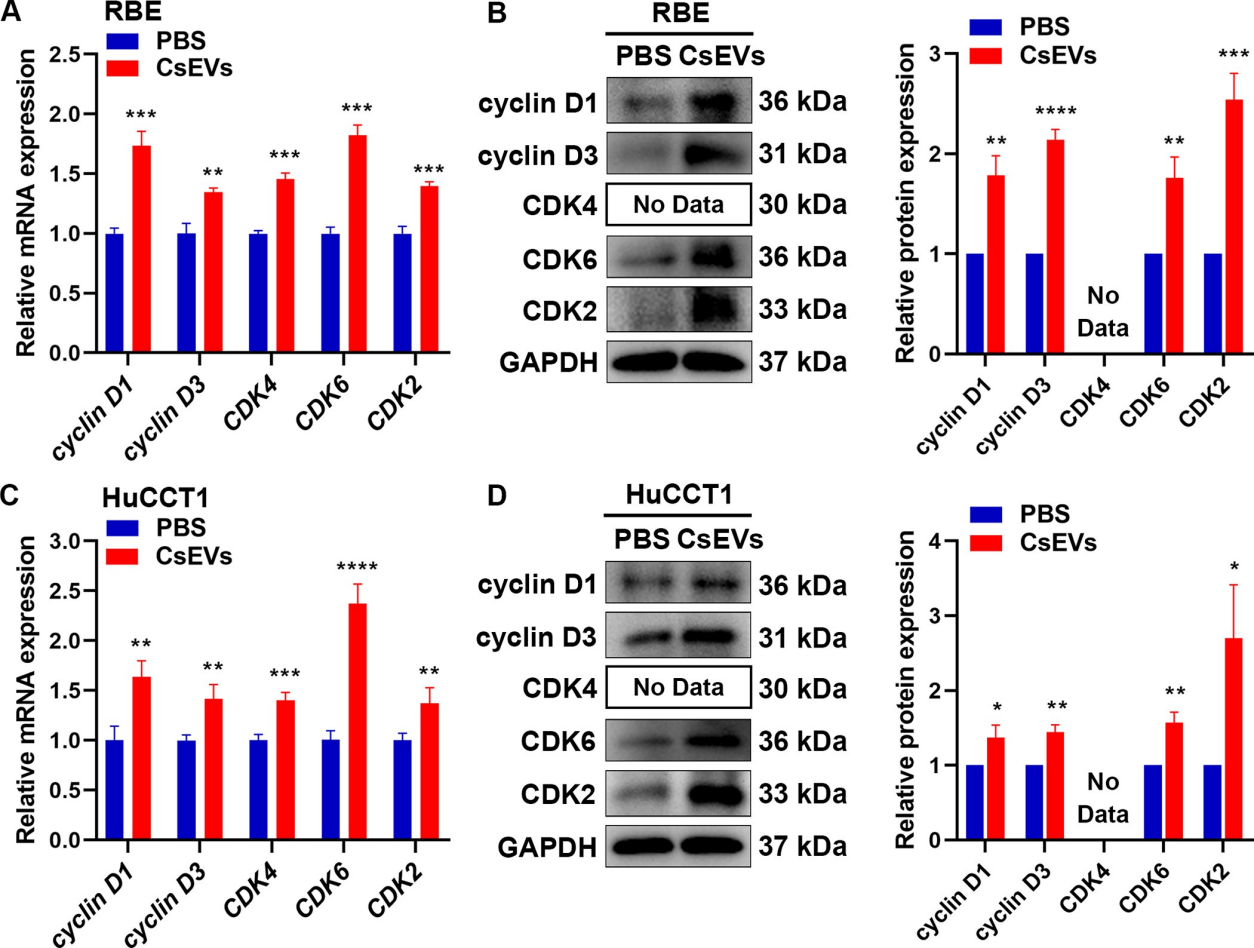
*Cs*EVs are involved in regulating the expression of cell cycle-related molecules. **(A, C)** The relative mRNA expression of cyclinD1, cyclinD3, CDK4, CDK6, and CDK2 were detected in RBE and HuCCT1 cells after treatment with or without 10 μg/ml of *Cs*EVs for 48 h using RT-qPCR. Equal volume PBS-treated group was used as negative control, and the mRNA fold change was normalized to β-actin. **(B, D)** The relative protein levels of cyclinD1, cyclinD3, CDK6, and CDK2 were detected in RBE and HuCCT1 cells after treatment with or without 10 μg/ml of *Cs*EVs for 48 h using western blot. Representative images were shown and the quantification of protein expression was measured by Image J and normalized to GAPDH. All experiments were conducted three times.

Cell cycle regulation is crucial for cell proliferation and growth, which is orchestrated by an intricate and delicate molecular network with the participation of multiple proteins, enzymes, cytokines, and cell cycle signaling pathways [25,26]. To shed light on the potential molecular mechanism of *Cs*EVs induced malignant proliferation in CCA cells, we examined the expression of various cell cycle-related regulators both at the transcriptional and translational levels. As shown in Fig 5, the relative mRNA and protein expression of cyclinD1 (RBE: RT-qPCR: *p* < 0.001, WB: *p* < 0.01; HuCCT1: RT-qPCR: *p* < 0.01, WB: *p* < 0.05), cyclinD3 (RBE: RT-qPCR: *p* < 0.01, WB: *p* < 0.0001; HuCCT1: RT-qPCR: *p* < 0.01, WB: *p* < 0.01), CDK4 (RBE: RT-qPCR: *p* < 0.001, WB: No data; HuCCT1: RT-qPCR: *p* < 0.001, WB: No data), CDK6 (RBE: RT-qPCR: *p* < 0.001, WB: *p* < 0.01; HuCCT1: RT-qPCR: *p* < 0.0001, WB: *p* < 0.01) and CDK2 (RBE: RT-qPCR: *p* < 0.001, WB: *p* < 0.001; HuCCT1: RT-qPCR: *p* < 0.01, WB: *p* < 0.05) were significantly increased in RBE and HuCCT1 cells following *Cs*EVs treatment, implying that *Cs*EVs can accelerate G1/S transition by regulating cell cycle-related molecules, thereby promoting the proliferation and oncogenicity of CCA cells.

### *Cs*EVs promote the migration, invasion, and lung metastasis of CCA cells

**Fig 6:**
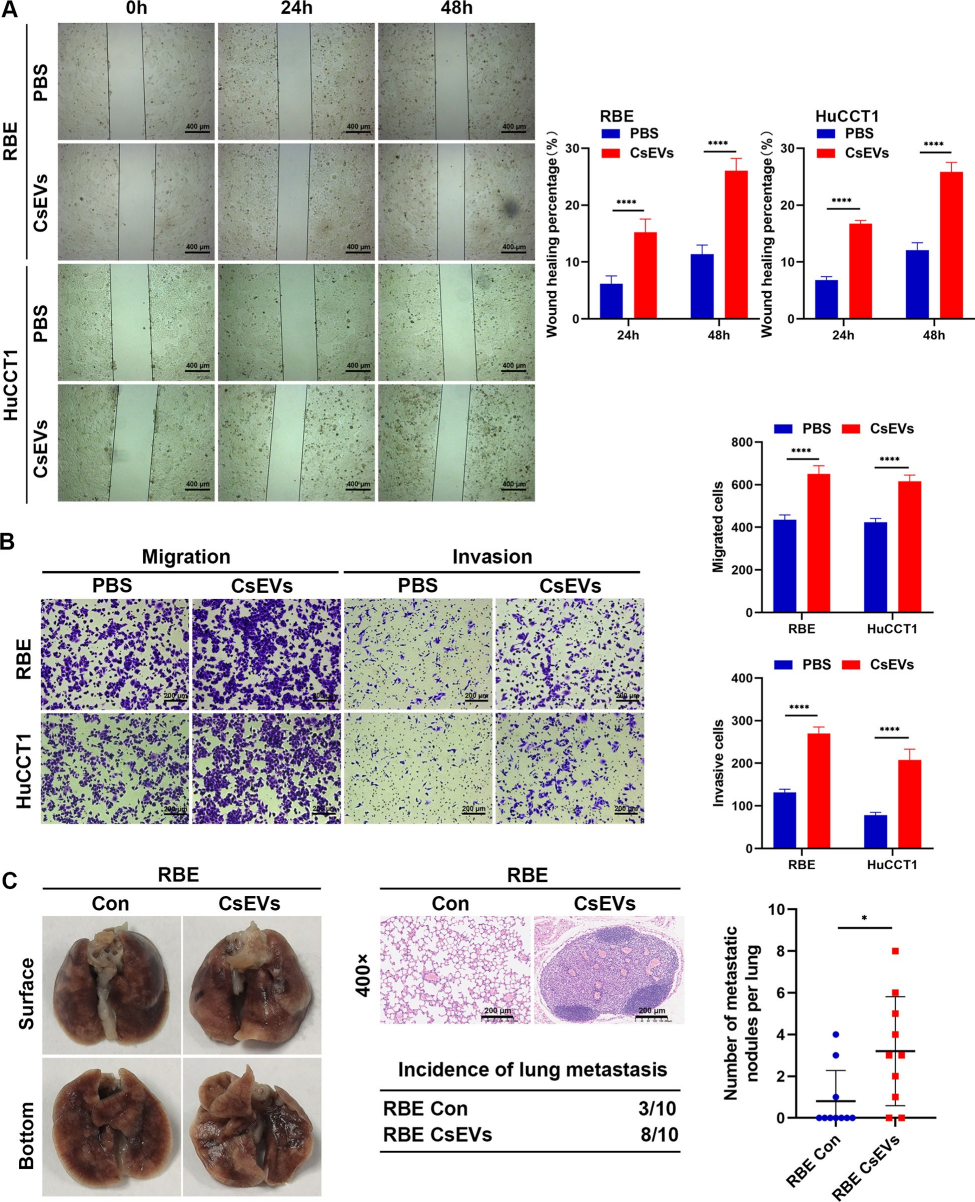
*Cs*EVs promote the migration and invasion of CCA cells. **(A)** Wound healing assay for RBE and HuCCT1 cells showed motility changes after treatment with or without 10 μg/ml of *Cs*EVs for 24 h. Images were taken at 0 h, 24 h, and 48 h after scratching, and representative pictures were shown. Scale Bar: 400 μm. **(B)** Transwell assay was conducted to determine the migration and invasion abilities of RBE and HuCCT1 cells after treatment with or without 10 μg/ml of *Cs*EVs for 24 h. Representative images of matrigel-free (for migration) and matrigel-coated (for invasion) transwell assay were shown. Three independent experiments were implemented for each group. Scale Bar: 200 μm. **(C)** The *in vivo* lung metastasis model. Representative images of specimens and H&E staining images of lung metastasis for the indicated RBE cells were shown. Table shows incidences of lung metastasis in the different groups. Histogram shows number of metastatic nodules in the different groups. Scale Bar: 200 μm.

We then detected whether *Cs*EVs could facilitate the migration and invasion of CCA cells *in vitro* and *in vivo*. Wound healing assays showed that *Cs*EVs significantly increased the 24-hour and 48-hour migration rates of RBE and HuCCT1 cells in comparison to the control group (*p* < 0.0001) (Fig 6A). Moreover, results of Transwell assays were consistent with those of the wound healing assays. Both matrigel-free (for migration) and matrigel-coated (for invasion) Transwell assays showed that the number of cells passing through the chamber within 24 h after *Cs*EVs treatment were markedly elevated (*p* < 0.0001) (Fig 6B). The lung metastasis model was established by injecting CCA cells into athymic nude mice through their tail veins to evaluate cell metastasis ability *in vivo*. We found that *Cs*EVs-treated RBE cells were more likely to increase the incidence of lung metastasis and the number of metastatic nodules than control cells (*p* < 0.05) (Fig 6C). Taken together, these experiments provide evidence that *Cs*EVs are involved in the malignancy of CCA by promoting cell metastasis.

### *Cs*EVs can induce EMT, and Slug is essential for *Cs*EVs-mediated EMT and CCA metastasis

**Fig 7:**
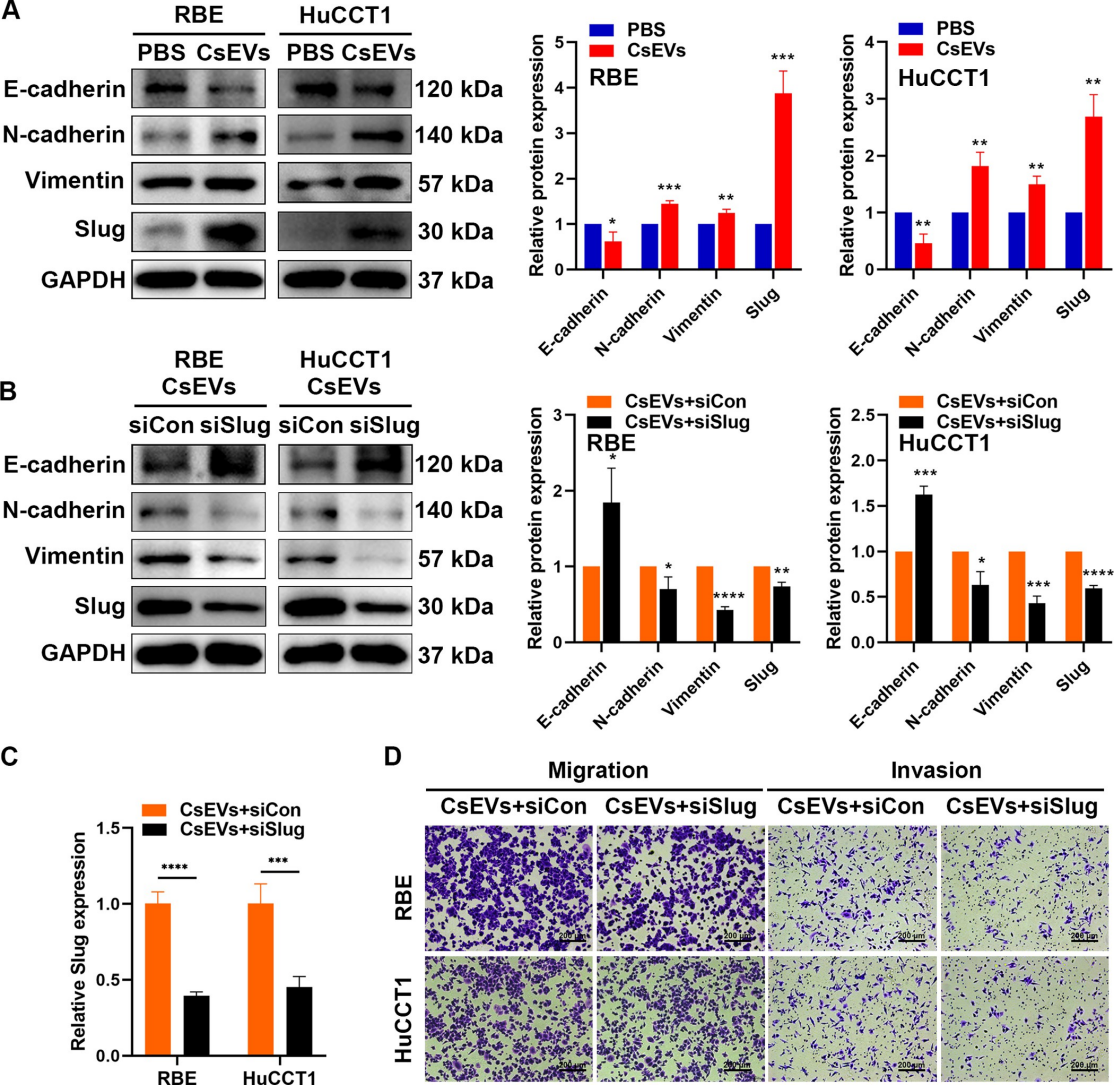
*Cs*EVs can induce EMT in CCA cells, and Slug is essential for *Cs*EVs -mediated EMT and CCA metastasis. **(A)** The expression of E-cadherin, N-cadherin, vimentin, and slug were detected in RBE and HuCCT1 cells after treatment with or without 10 μg/ml of *Cs*EVs for 48 h by western blot. Representative images were shown and the quantification of protein expression was measured by Image J and normalized to GAPDH. **(B)** RBE and HuCCT1 cells were pretreated with 10 μg/ml of *Cs*EVs for 24 h, followed by transfection with Slug siRNA for 48 h. siCon was used as a transcriptional negative control. Western blot showed that Slug knockdown reversed the levels of the EMT-related proteins affected by *Cs*EVs treatment. **(C)** The relative mRNA expression of Slug was measured in transfected CCA cells using RT-qPCR, and the mRNA fold change was normalized to β-actin. **(D)** After the indicated treatment, representative images of transwell assays demonstrated that downregulated Slug reversed the number of migrated and invasive CCA cells pretreated with *Cs*EVs. All experiments were repeated three times. Scale Bar: 200 μm.

EMT is a key process by which tumor cells acquire highly migratory and invasive phenotypic characteristics [27], so we tried to investigate the correlation between *Cs*EVs and EMT in CCA. Western blot assays were performed to analyze the effect of *Cs*EVs on EMT-related markers in RBE and HuCCT1 cells, and it was revealed that the expression of E-cadherin (an epithelial type marker) was decreased followed by *Cs*EVs treatment, whereas the levels of N-cadherin and vimentin (mesenchymal type markers) was up-regulated. Concurrently, *Cs*EVs overexpression in RBE and HuCCT1 cells significantly increased (RBE: *p* < 0.001, HuCCT1: *p* < 0.01) the expression of Slug, one of the most important EMT-associated transcription factors that can participate in the regulation of EMT markers [28] (Fig 7A). We hypothesized that Slug might play an important role in *Cs*EVs-mediated EMT progression, and therefore, siRNA was used to specifically knock down Slug. The results indicated that the expression of Slug was decreased at both transcriptional and translational levels after transfection with Slug siRNA (Fig 7B and 7C), while the expression of E-cadherin was restored and N-cadherin as well as vimentin had an inverse expression pattern in the transfected RBE and HuCCT1 cells (Fig 7B). Consistent conclusions were obtained by transwell assays. Knockdown of Slug suppressed the migration and invasion of *Cs*EVs treated RBE and HuCCT1 cells (Figs 7D and S1). Together, these results indicate that *Cs*EVs can induce EMT process, and Slug is crucial for *Cs*EVs-mediated EMT and metastasis in CCA.

### *Cs*EVs promote EMT and malignant phenotypes of CCA cell lines through activating the NF-κB signaling pathway

**Fig 8:**
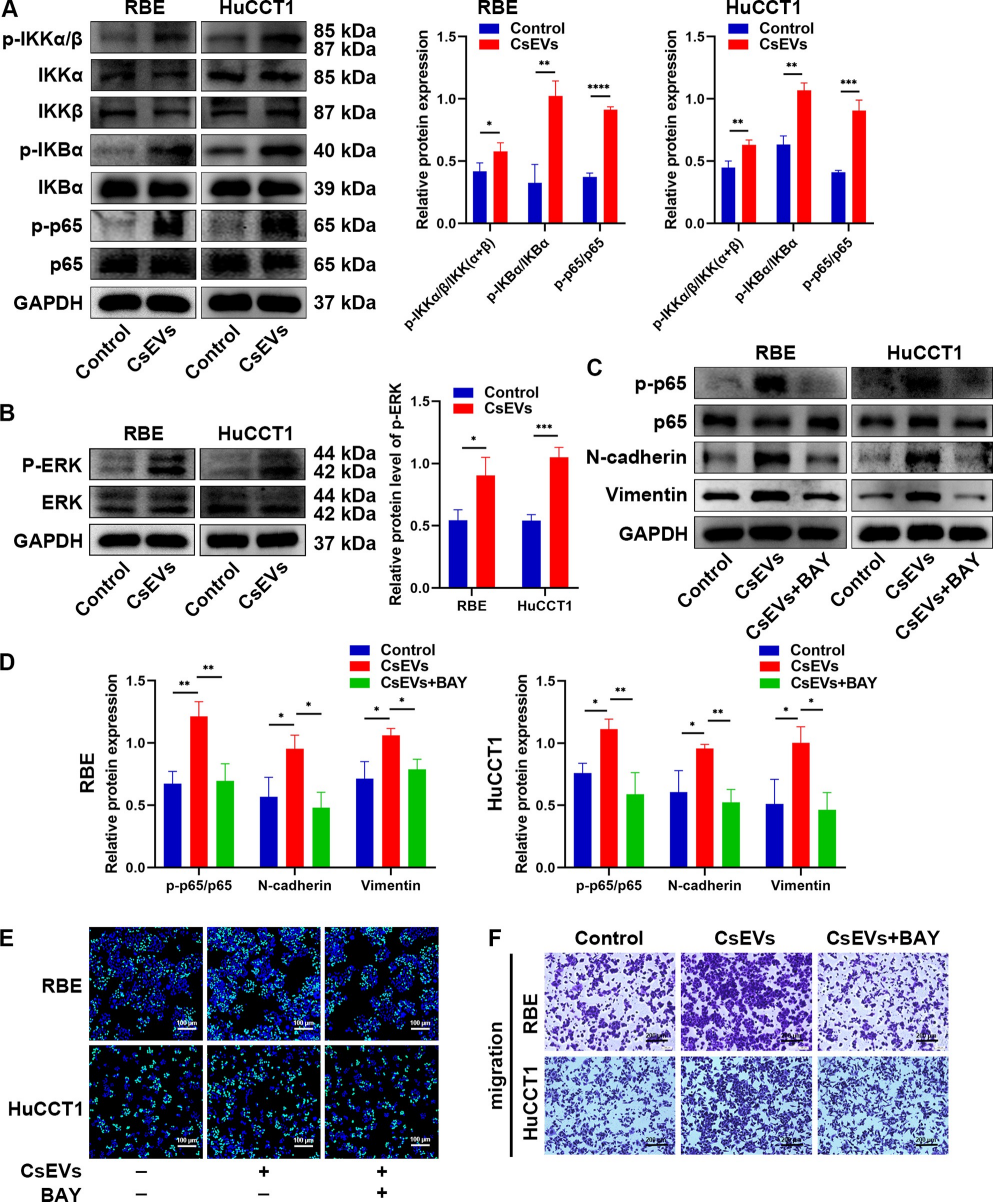
The NF-κB and ERK signaling pathways were involved in *Cs*EVs- mediated EMT and malignant phenotypes. **(A)** The expression of key proteins in NF-κB pathway were detected in RBE and HuCCT1 cells after treatment with or without 10 μg/ml of *Cs*EVs for 48 h by western blot. Representative images were shown and the quantification of protein expression was measured by Image J. **(B)** The phosphorylation of ERK was determined by western blot after the indicated treatment. **(C, D)** RBE and HuCCT1 cells were pretreated with 10 μM BAY 11–7082 (a NF-κB inhibitor) for 1 h, followed by stimulation with 10 μg/ml of *Cs*EVs for 24 h. Then, western blot assay was conducted to evaluate the expression levels of p-p65/p65, N-cadherin and vimentin. **(E)** EdU-488 assay and **(F)** Transwell assay were used to determine the proliferation and migration ability of RBE and HuCCT1 cells after treatment with NF-κB inhibitor. All assays were executed for three times.

To further explore the potential mechanism of *Cs*EVs-induced alterations in CCA cells, we examined the NF-κB and ERK pathways using western blot analysis, which might be involved in the regulation of EMT and tumor metastasis [13,29]. As shown in Fig 8A, the phosphorylation of IκB kinase α/β (p-IKKα/β), IκB-α (p-IκB-α) and p65 (p-p65), some essential molecules in the activation of the NF-κB signaling pathway, were considerably elevated in RBE and HuCCT1 cells after *Cs*EVs stimulation, especially p-p65. Also, the phosphorylation of ERK (p-ERK) was simultaneously upregulated (Fig 8B). These results demonstrated that *Cs*EVs activated the NF-κB and ERK signaling pathways by promoting protein phosphorylation. Given that the NF-κB/EMT axis was found to participate in facilitating metastasis of cancer cells [30], we pondered whether the NF-κB pathway indeed regulated the malignant phenotypes of CCA via its impact on EMT mechanism. BAY 11–7082, a specific NF-κB inhibitor, was employed to pretreat RBE and HuCCT1 cells for 1 h, followed by *Cs*EVs treatment. Results showed that NF-κB inhibitor could impede the phosphorylation of p65 and decrease the expression levels of N-cadherin and vimentin upregulated by *Cs*EVs (Fig 8C and 8D). Furthermore, EdU-488 and Transwell assays revealed that NF-κB inhibitor markedly reversed the *Cs*EVs-induced malignant proliferation and migration in both RBE and HuCCT1 cells (Fig 8E and 8F and S2 Fig). Taken together, these findings suggested that *Cs*EVs accelerated malignant proliferation, migration and invasion of CCA cells through NF-κB/EMT axis.

## Discussion

**Fig 9:**
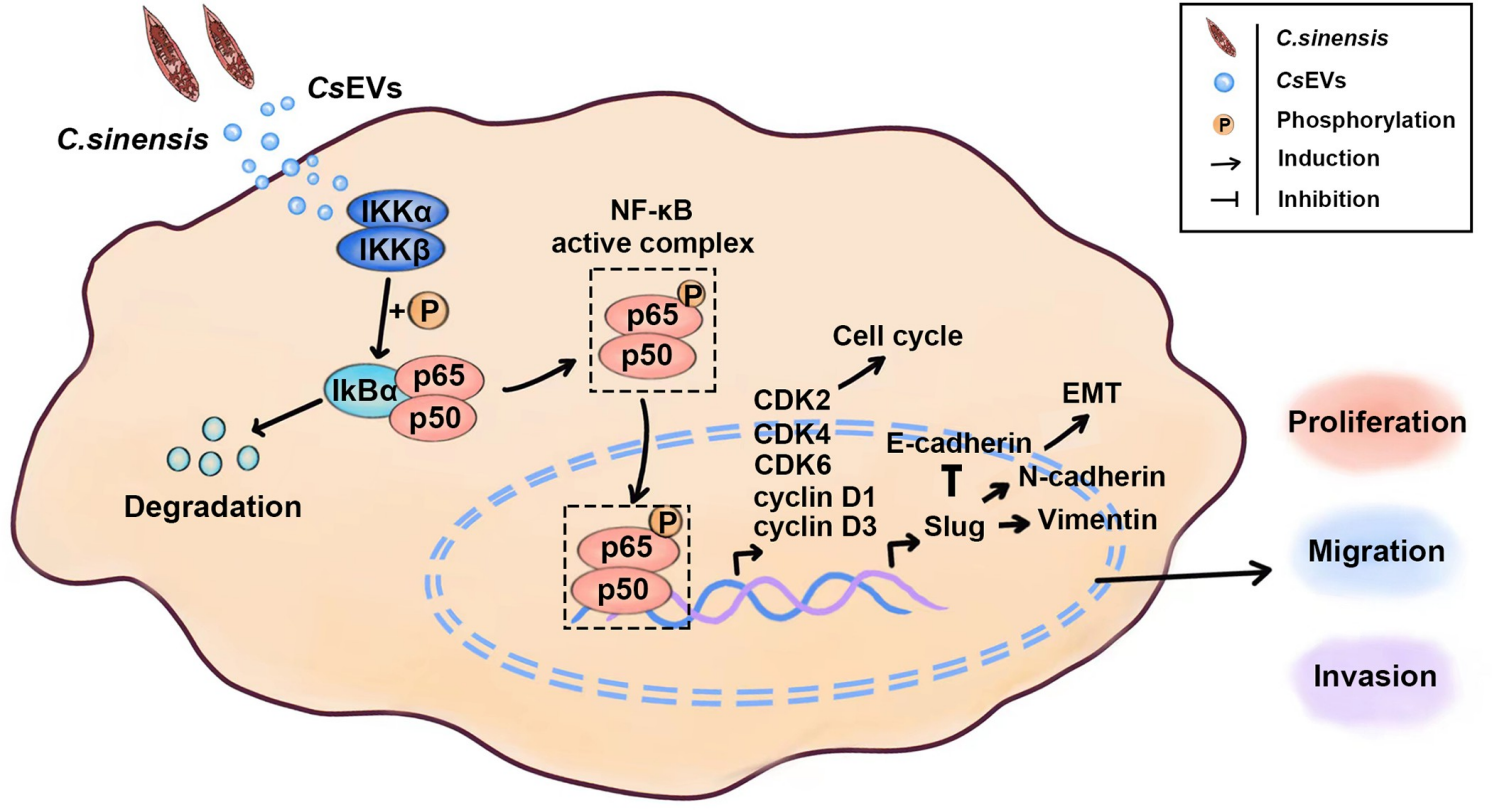
Schematic diagram of *Cs*EVs in promoting malignant proliferation and metastasis of CCA cells. EVs isolated from the culture supernatant of *C*. *sinensis* adult worms could facilitate the abnormal proliferation of CCA cells by regulating cell cycle progression, and promote the migration, invasion and EMT process through activating the NF-κB signaling pathway.

In endemic areas, chronic liver fluke infection is thought to be a significant initiator of CCA [31]. Clinical statistics showed that the patients with CCA complicated by *C*. *sinensis* infection tend to be younger than those with CCA alone, and those under the age of 64 had a worse overall survival rate [32]. So far, the exact mechanism by which *C*. *sinensis* infection drives CCA to proceed to malignancy has not been established. Parasite-secreted EVs have been reported to affect the normal physiological function of recipient cells by transferring proteins, lipids, and nucleic acids in parasite-host interactions, leading to the pathogenesis of host [16]. For example, it has been shown that *Cs*EVs can promote bile duct damage and inflammation, which are the important stages before carcinogenesis [17,19]. Numerous studies have shown a clear connection between EVs and tumor progression [33,34]. However, studies on the associations between *Cs*EVs and CCA are limited. Therefore, further research into the specific mechanisms through which *Cs*EVs affect the malignant phenotypes of CCA is required. In the present study, it was discovered that EVs isolated from the culture supernatant of *C*. *sinensis* adult worms could facilitate the abnormal proliferation of CCA cells by regulating cell cycle progression, and promote the migration, invasion and EMT process through activating the NF-κB signaling pathway (Fig 9).

Different EVs separation techniques will generate high heterogeneity of their concentration and purity, which may yield discrepant research results [35]. Considering that helminth-derived EVs have been successfully separated using the gold standard approach of differential ultracentrifugation [17,20], we referred to these protocols with slight adjustments to enrich *Cs*EVs. The isolated *Cs*EVs appeared to have a cup-like or circular form with a peak size of 79 nm, which was compatible with the defining characteristics of EVs [23]. Furthermore, our findings provided the first insight into the protein expression profile in *Cs*EVs. In comparison with the proteins enriched in EVs, there are extensive match [23]. Also, some proteins have been identified in *Cs*ESPs previously [22], which deserve further research. For instance, myoferlin, a membrane protein of ferlin family, has been reported to be involved in tumor progression by promoting angiogenesis, energy metabolism reprogramming, EMT and exosome modulation, which may be a potential target for tumor detection and therapy [36]. These results support that we have successfully obtained *Cs*EVs.

PKH26 is a lipophilic fluorescent dye that can specifically label exosomes by interacting with the lipid bilayer of the membrane structure [37]. We used PKH26-labeled *Cs*EVs to detect the uptake ability of CCA cells, and the results illustrated that *Cs*EVs could be captured and functioned inside the cells. To discover the pathophysiologic significance and underlying mechanism of *Cs*EVs in CCA, a series of experiments were performed, which showed that the proliferation, migration and invasion abilities of CCA cells were enhanced after treatment with *Cs*EVs. To investigate the possible mechanism by which *Cs*EVs promote proliferation, we examined the cell cycle distribution and expression levels of related regulators, as a disorder of the cell cycle can cause malignant proliferation and ultimately contribute to cancer initiation and progression [26]. The results suggested that *Cs*EVs could accelerate G1/S transition to expedite cell proliferation by increasing the expression of positive cell cycle regulators. However, we did not delve into the detailed mechanisms of cell cycle dysregulation brought on by *Cs*EVs, and we hope to explore this aspect in future studies.

Malignant tumor cells generated from epithelium go through a critical biological process called EMT to gain the capacity for invasion and migration, including CCA [9]. We hypothesized that *Cs*EVs may promote CCA metastasis by inducing the EMT process, and we demonstrated that *Cs*EVs could affect the expression of EMT-related markers at the transcriptional and translational levels. The decrease of E-cadherin can destroy the tight junction between cells, which is conducive to cell metastasis. Meanwhile, the up-regulation of N-cadherin and vimentin also mediate cell adhesion and migration. Most importantly, we discovered that Slug, one of the most significant EMT-associated transcription factors that can regulate EMT markers [28], was dramatically upregulated after stimulation with *Cs*EVs. When being transfected with siRNA to knock down Slug, the EMT process was partially reversed, hinting that Slug may be an important target molecule in *Cs*EVs-mediated EMT.

NF-κB is an essential intracellular nuclear transcription factor, which participates in a variety of physiological and pathological processes such as inflammation, immune response, cell survival and tumorigenesis [29]. Moreover, the interaction between the NF-κB pathway and the EMT process has been reported in various cancers, showing that NF-κB can act as an upstream mediator to induce EMT for expediting tumor metastasis by collaborating with multiple other signaling molecules and pathways. NF-κB/EMT axis could be a critical way in triggering the malignant progression of tumor cells [30]. Thus, we concentrated on the interaction between NF-κB signaling pathway and *Cs*EVs-mediated EMT. Here, it was demonstrated that the phosphorylation of key molecules in the NF-κB pathway was provoked by *Cs*EVs, and its downstream effects were crucial for the occurrence of EMT and malignant metastasis in CCA. Previous studies have reported that ERK pathway is involved in inducing abnormal proliferation, invasive growth, and distant metastasis of tumors [38]. We currently found the phosphorylation of ERK, but further studies are requisite to verify the role of ERK pathway in *Cs*EVs-induced CCA malignant phenotypes. In addition, we did not perform enough *in vivo* experiments to corroborate these findings. Therefore, future research will explore the role of *Cs*EVs in the malignant phenotypes and tumor microenvironment of CCA by constructing a tumor-bearing mouse model. Moreover, elucidation of exactly which proteins in *Cs*EVs are acting to activate such a broad range of signaling pathways is needed.

In conclusion, our study provides evidence that *Cs*EVs can induce the malignant progression of CCA cells by promoting proliferation, migration and invasion via NF-κB/EMT axis. Meanwhile, the protein expression profile of *Cs*EVs is revealed by this study, which provides a theoretical basis for understanding the function of *Cs*EVs and proposes a novel mechanism to explain the liver fluke-associated CCA.

## Supporting information

S1 Fig The quantification of migrated and invasive CCA cells after *Cs*EVs treatment and Slug knockdown. RBE and HuCCT1 cells were pretreated with 10 μg/ml of *Cs*EVs for 24 h, followed by transfection with Slug siRNA for 48 h. siCon was used as a transcriptional negative control. Transwell assays demonstrated that downregulated Slug reversed the number of migrated **(A)** and invasive **(B)** CCA cells.
(DOCX)

S2 Fig The quantification of EdU-488 assays and transwell assays after the employment of NF-κB inhibitor. RBE and HuCCT1 cells were pretreated with 10 μM BAY 11–7082 (a NF-κB inhibitor) for 1 h, followed by stimulation with 10 μg/ml of *Cs*EVs for 24 h. EdU-488 assays **(A, B)** and Transwell assays **(C, D)** revealed that NF-κB inhibitor markedly reversed the *Cs*EVs-induced malignant proliferation and migration in both RBE and HuCCT1 cells.
(DOCX)

S1 Table siRNA for transfection. (DOCX)

S2 Table Primer sequences of human genes used in RT-qPCR. (DOCX)

S3 Table Specifications of primary antibodies. (DOCX)

S4 Table The information of the identified *Cs*EVs proteins. (XLSX)

S1 File Raw data and descriptive statistics. (DOCX)
