# Supplementary material for: Extracellular vesicles of Clonorchis sinensis promote the malignant phenotypes of cholangiocarcinoma via NF-κB/EMT axis
Source: PLoS Negl Trop Dis. 2024 Oct 28;18(10):e0012545. doi: 10.1371/journal.pntd.0012545 (PMC11516169; doi:10.1371/journal.pntd.0012545)
Supplement: S1 Fig — RBE and HuCCT1 cells were pretreated with 10 μg/ml of CsEVs for 24 h, followed by transfection with Slug siRNA for 48 h. siCon was used as a transcriptional negative control. Transwell assays demonstrated that downregulated Slug reversed the number of migrated (A) and invasive (B) CCA cells. (DOCX) [file pntd.0012545.s001.docx]

**S1 Fig. The quantification of migrated and invasive CCA cells after *Cs*EVs treatment and Slug knockdown.** RBE and HuCCT1 cells were pretreated with 10 μg/ml of *Cs*EVs for 24 h, followed by transfection with Slug siRNA for 48 h. siCon was used as a transcriptional negative control. Transwell assays demonstrated that downregulated Slug reversed the number of migrated **(A)** and invasive **(B)** CCA cells.

**
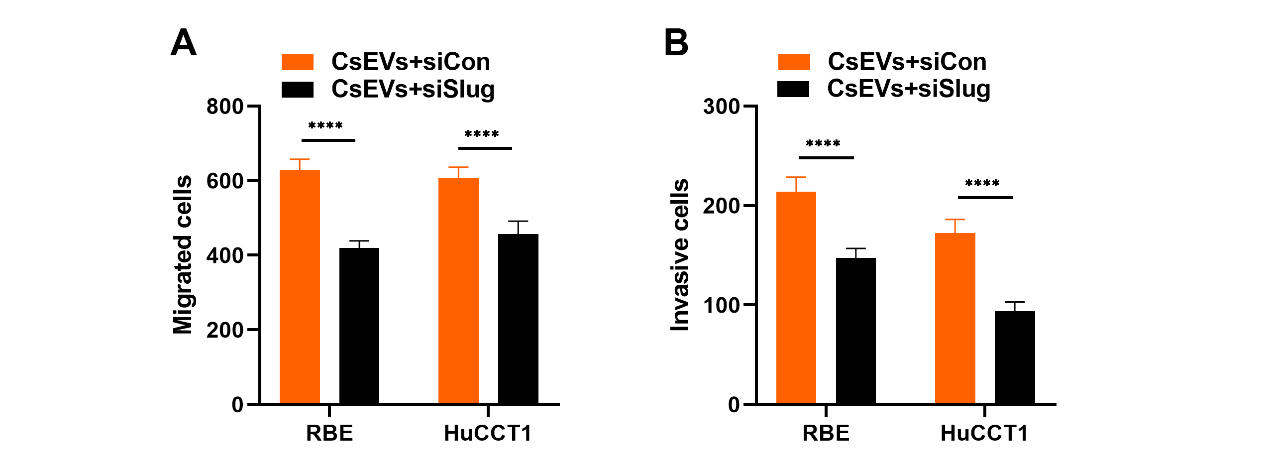
**
