# Supplementary material for: Extracellular vesicles of Clonorchis sinensis promote the malignant phenotypes of cholangiocarcinoma via NF-κB/EMT axis
Source: PLoS Negl Trop Dis. 2024 Oct 28;18(10):e0012545. doi: 10.1371/journal.pntd.0012545 (PMC11516169; doi:10.1371/journal.pntd.0012545)

**S2 Fig. The quantification of EdU-488 assays and transwell assays after the employment of NF-κB inhibitor.** RBE and HuCCT1 cells were pretreated with 10 μM BAY 11-7082 (a NF-κB inhibitor) for 1 h, followed by stimulation with 10 μg/ml of *Cs*EVs for 24 h. EdU-488 assays **(A, B)** and Transwell assays **(C, D)** revealed that NF-κB inhibitor markedly reversed the *Cs*EVs-induced malignant proliferation and migration in both RBE and HuCCT1 cells.


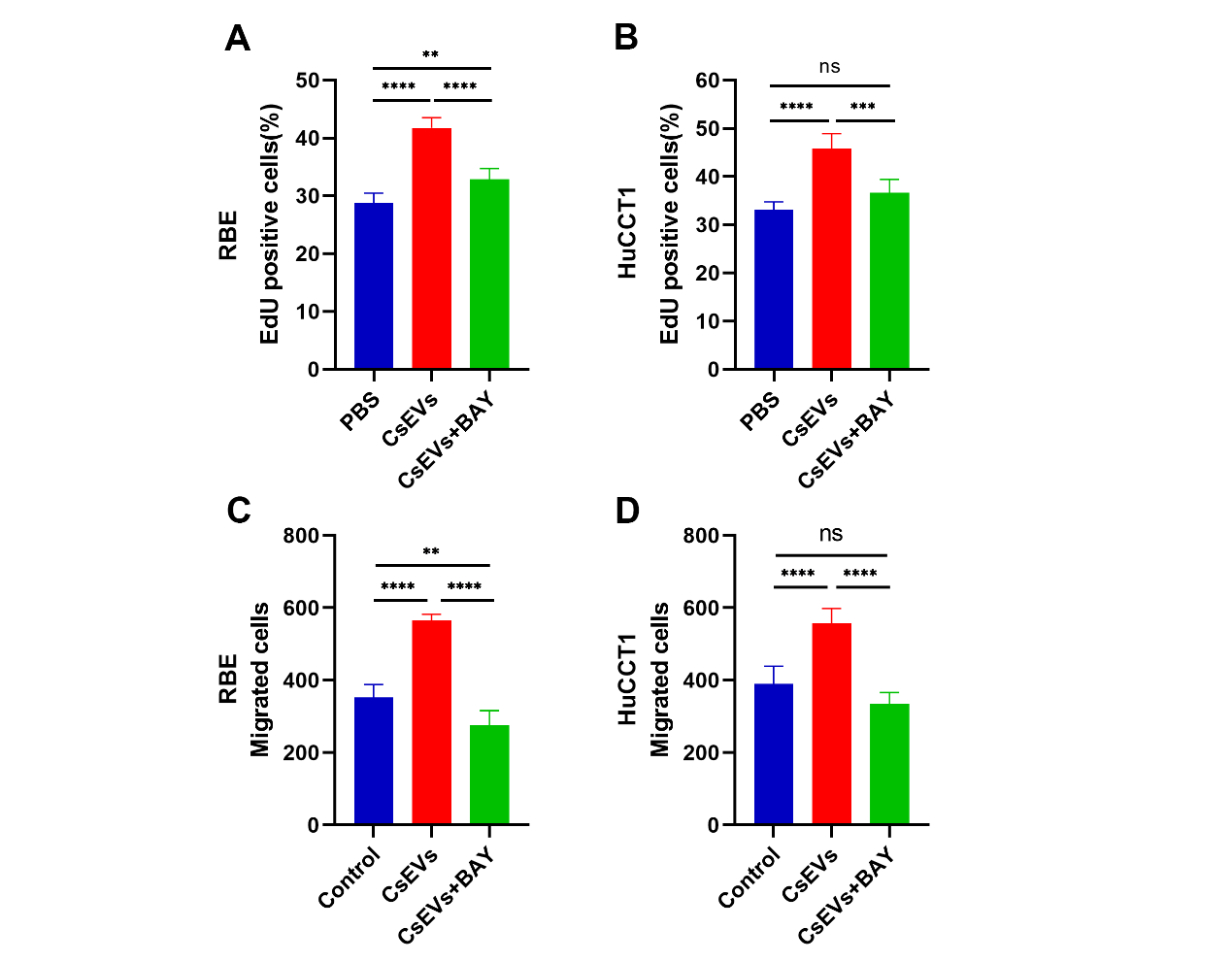

Supplement: S2 Fig — RBE and HuCCT1 cells were pretreated with 10 μM BAY 11–7082 (a NF-κB inhibitor) for 1 h, followed by stimulation with 10 μg/ml of CsEVs for 24 h. EdU-488 assays (A, B) and Transwell assays (C, D) revealed that NF-κB inhibitor markedly reversed the CsEVs-induced malignant proliferation and migration in both RBE and HuCCT1 cells. (DOCX) [file pntd.0012545.s002.docx]
