# Supplementary material for: Extracellular vesicles of Clonorchis sinensis promote the malignant phenotypes of cholangiocarcinoma via NF-κB/EMT axis
Source: PLoS Negl Trop Dis. 2024 Oct 28;18(10):e0012545. doi: 10.1371/journal.pntd.0012545 (PMC11516169; doi:10.1371/journal.pntd.0012545)
Supplement: S1 Table — (DOCX) [file pntd.0012545.s003.docx]

**S1 Table. siRNA for transfection**

| **siRNA (gene)** | **Sequence** | |
| --- | --- | --- |
|  | **sense (5’-3’)** | **Antisense (5’-3’)** |
| HS-Slug | CAAAUCAUUUCAACUGAAATT | UUUCAGUUGAAAUGAUUUGTT |
| HS-control | UUCUCCGAACGUGUCACGUTT | ACGUGACACGUUCGGAGAATT |
