# Supplementary material for: Extracellular vesicles of Clonorchis sinensis promote the malignant phenotypes of cholangiocarcinoma via NF-κB/EMT axis
Source: PLoS Negl Trop Dis. 2024 Oct 28;18(10):e0012545. doi: 10.1371/journal.pntd.0012545 (PMC11516169; doi:10.1371/journal.pntd.0012545)
Supplement: S2 Table — (DOCX) [file pntd.0012545.s004.docx]

**S2 Table. Primer sequences of human genes for RT-qPCR**

| **Genes** | **Forward (5’-3’)** | **Reverse (5’-3’)** |
| --- | --- | --- |
| cyclin D1 | GCTGCGAAGTGGAAACCATC | CCTCCTTCTGCACACATTTGAA |
| cyclin D3 | TACCCGCCATCCATGATCG | AGGCAGTCCACTTCAGTGC |
| CDK2 | CCAGGAGTTACTTCTATGCCTGA | TTCATCCAGGGGAGGTACAAC |
| CDK4 | ATGGCTACCTCTCGATATGAGC | CATTGGGGACTCTCACACTCT |
| CDK6 | GCTGACCAGCAGTACGAATG | GCACACATCAAACAACCTGACC |
| Slug | CGAACTGGACACACATACAGTG | CTGAGGATCTCTGGTTGTGGT |
| β-actin | CTGGCACCACACCTTCTACAATG | AATGTCACGCACGATTTCCCGC |
