# Supplementary material for: Extracellular vesicles of Clonorchis sinensis promote the malignant phenotypes of cholangiocarcinoma via NF-κB/EMT axis
Source: PLoS Negl Trop Dis. 2024 Oct 28;18(10):e0012545. doi: 10.1371/journal.pntd.0012545 (PMC11516169; doi:10.1371/journal.pntd.0012545)
Supplement: S3 Table — (DOCX) [file pntd.0012545.s005.docx]

**S3 Table. Specifications of primary antibodies**

| Vendor | Antibody | Source | Catalog no. | Working dilution |
| --- | --- | --- | --- | --- |
| Proteintech | anti-E-cadherin | Mouse | 60335-1-Ig | WB, 1:2000 |
| Cell Signaling Technology | anti-N-cadherin | Rabbit | 13116 | WB, 1:1000 |
|  | anti-Vimentin | Rabbit | 5741 | WB, 1:1000 |
|  | anti-Slug | Rabbit | 9585 | WB, 1:1000 |
|  | anti-CDK2 | Rabbit | 18048 | WB, 1:1000 |
|  | anti-CDK6 | Mouse | 3136 | WB, 1:2000 |
|  | anti-CyclinD1 | Rabbit | 55506 | WB, 1:1000 |
|  | anti-CyclinD3 | Mouse | 2936 | WB, 1:2000 |
|  | anti-p-ERK | Rabbit | 4370 | WB, 1:2000 |
|  | anti-ERK | Rabbit | 4695 | WB, 1:1000 |
|  | anti-p-IKKα/β | Rabbit | 2697 | WB, 1:1000 |
|  | anti-IKKα | Mouse | 11930 | WB, 1:1000 |
|  | anti-IKKβ | Rabbit | 8943 | WB, 1:1000 |
|  | anti-p-IKBα | Rabbit | 2859 | WB, 1:1000 |
|  | anti-IKBα | Mouse | 4814 | WB, 1:1000 |
|  | anti-p-p65 | Rabbit | 3033 | WB, 1:1000 |
|  | anti-p65 | Rabbit | 8242 | WB, 1:1000 |
|  | anti-GAPDH | Rabbit | 5174 | WB, 1:1000 |
