# Supplementary material for: Extracellular vesicles of Clonorchis sinensis promote the malignant phenotypes of cholangiocarcinoma via NF-κB/EMT axis
Source: PLoS Negl Trop Dis. 2024 Oct 28;18(10):e0012545. doi: 10.1371/journal.pntd.0012545 (PMC11516169; doi:10.1371/journal.pntd.0012545)
Supplement: S1 File — (DOCX) [file pntd.0012545.s007.docx]

**Raw data and statistical analysis of Figure 4:**

**CCK8: RBE**

| **Time (h)** | **0μg/ml** | | | | | **5μg/ml** | | | | |
| --- | --- | --- | --- | --- | --- | --- | --- | --- | --- | --- |
| **0** | 0.341 | 0.337 | 0.339 | 0.339 | 0.322 | 0.339 | 0.365 | 0.367 | 0.358 | 0.333 |
| **24** | 0.534 | 0.499 | 0.58 | 0.502 | 0.523 | 0.599 | 0.599 | 0.603 | 0.609 | 0.635 |
| **48** | 0.638 | 0.644 | 0.672 | 0.66 | 0.65 | 0.695 | 0.711 | 0.7 | 0.688 | 0.689 |
| **72** | 0.798 | 0.848 | 0.865 | 0.852 | 0.817 | 0.903 | 0.869 | 0.862 | 0.872 | 0.878 |

| **Time (h)** | **10μg/ml** | | | | | **20μg/ml** | | | | |
| --- | --- | --- | --- | --- | --- | --- | --- | --- | --- | --- |
| **0** | 0.334 | 0.337 | 0.33 | 0.337 | 0.304 | 0.323 | 0.365 | 0.374 | 0.358 | 0.303 |
| **24** | 0.824 | 0.798 | 0.811 | 0.844 | 0.839 | 0.724 | 0.716 | 0.761 | 0.714 | 0.725 |
| **48** | 1.314 | 1.386 | 1.438 | 1.404 | 1.36 | 0.963 | 0.985 | 0.971 | 0.995 | 0.961 |
| **72** | 1.4 | 1.444 | 1.47 | 1.434 | 1.464 | 0.788 | 0.795 | 0.769 | 0.774 | 0.758 |

**CCK8: HuCCT1**

| **Time (h)** | **0μg/ml** | | | | | **5μg/ml** | | | | |
| --- | --- | --- | --- | --- | --- | --- | --- | --- | --- | --- |
| **0** | 0.279 | 0.284 | 0.291 | 0.286 | 0.284 | 0.298 | 0.269 | 0.278 | 0.282 | 0.294 |
| **24** | 0.368 | 0.374 | 0.375 | 0.369 | 0.368 | 0.41 | 0.425 | 0.406 | 0.417 | 0.425 |
| **48** | 0.54 | 0.542 | 0.557 | 0.546 | 0.549 | 0.569 | 0.59 | 0.608 | 0.563 | 0.58 |
| **72** | 0.703 | 0.688 | 0.711 | 0.695 | 0.697 | 0.717 | 0.721 | 0.761 | 0.722 | 0.725 |

| **Time (h)** | **10μg/ml** | | | | | **20μg/ml** | | | | |
| --- | --- | --- | --- | --- | --- | --- | --- | --- | --- | --- |
| **0** | 0.29 | 0.276 | 0.266 | 0.289 | 0.287 | 0.294 | 0.276 | 0.271 | 0.261 | 0.281 |
| **24** | 0.62 | 0.668 | 0.657 | 0.667 | 0.685 | 0.545 | 0.557 | 0.565 | 0.562 | 0.564 |
| **48** | 0.997 | 0.971 | 0.981 | 0.968 | 0.971 | 0.702 | 0.714 | 0.702 | 0.718 | 0.688 |
| **72** | 1.276 | 1.286 | 1.289 | 1.246 | 1.248 | 0.621 | 0.623 | 0.629 | 0.629 | 0.613 |

**EdU positive cells(%)****: RBE**

| PBS | CsEVs |
| --- | --- |
| 29.67 | 42.06 |
| 26.64 | 42.13 |
| 28.96 | 44.37 |
| 32.09 | 40.99 |
| 32.88 | 40.8 |

| **Test for normal distribution** |  |  |
| --- | --- | --- |
| Shapiro-Wilk test | PBS | CsEVs |
| W | 0.9565 | 0.8685 |
| P value | 0.7833 | 0.2604 |
| Passed normality test (alpha=0.05)? | Yes | Yes |
| P value summary | ns | ns |
|  |  |  |
| Kolmogorov-Smirnov test | PBS | CsEVs |
| KS distance | 0.1924 | 0.2832 |
| P value | >0.1000 | >0.1000 |
| Passed normality test (alpha=0.05)? | Yes | Yes |
| P value summary | ns | ns |

| Column B | CsEVs |
| --- | --- |
| vs. | vs. |
| Column A | PBS |
|  |  |
| Unpaired t test |  |
| P value | <0.0001 |
| P value summary | **** |
| Significantly different (P < 0.05)? | Yes |
| One- or two-tailed P value? | Two-tailed |
| t, df | t=9.330, df=8 |
|  |  |
| How big is the difference? |  |
| Mean of column A | 30.05 |
| Mean of column B | 42.07 |
| Difference between means (B - A) ± SEM | 12.02 ± 1.288 |
| 95% confidence interval | 9.051 to 14.99 |
| R squared (eta squared) | 0.9158 |
|  |  |
| F test to compare variances |  |
| F, DFn, Dfd | 3.113, 4, 4 |
| P value | 0.2972 |
| P value summary | ns |
| Significantly different (P < 0.05)? | No |

**EdU positive cells(%): HuCCT1**

| PBS | CsEVs |
| --- | --- |
| 31.84 | 43.87 |
| 35.19 | 46.14 |
| 32.38 | 49.94 |
| 32.43 | 49.51 |
| 33.01 | 47.66 |

| **Test for normal distribution** |  |  |
| --- | --- | --- |
| Shapiro-Wilk test | PBS | CsEVs |
| W | 0.8264 | 0.9394 |
| P value | 0.1306 | 0.6616 |
| Passed normality test (alpha=0.05)? | Yes | Yes |
| P value summary | ns | ns |
|  |  |  |
| Kolmogorov-Smirnov test | PBS | CsEVs |
| KS distance | 0.2878 | 0.1979 |
| P value | >0.1000 | >0.1000 |
| Passed normality test (alpha=0.05)? | Yes | Yes |
| P value summary | ns | ns |

| Column B | CsEVs |
| --- | --- |
| vs. | vs. |
| Column A | PBS |
|  |  |
| Unpaired t test |  |
| P value | <0.0001 |
| P value summary | **** |
| Significantly different (P < 0.05)? | Yes |
| One- or two-tailed P value? | Two-tailed |
| t, df | t=11.45, df=8 |
|  |  |
| How big is the difference? |  |
| Mean of column A | 32.97 |
| Mean of column B | 47.42 |
| Difference between means (B - A) ± SEM | 14.45 ± 1.262 |
| 95% confidence interval | 11.54 to 17.36 |
| R squared (eta squared) | 0.9425 |
|  |  |
| F test to compare variances |  |
| F, DFn, Dfd | 3.654, 4, 4 |
| P value | 0.2373 |
| P value summary | ns |
| Significantly different (P < 0.05)? | No |

**Clone number****: RBE**

| PBS | CsEVs |
| --- | --- |
| 437 | 852 |
| 610 | 938 |
| 645 | 840 |

| **Test for normal distribution** |  |  |
| --- | --- | --- |
| Shapiro-Wilk test | PBS | CsEVs |
| W | 0.8720 | 0.8403 |
| P value | 0.3014 | 0.2148 |
| Passed normality test (alpha=0.05)? | Yes | Yes |
| P value summary | ns | ns |

| Column B | CsEVs |
| --- | --- |
| vs. | vs. |
| Column A | PBS |
|  |  |
| Unpaired t test |  |
| P value | 0.0118 |
| P value summary | * |
| Significantly different (P < 0.05)? | Yes |
| One- or two-tailed P value? | Two-tailed |
| t, df | t=4.384, df=4 |
|  |  |
| How big is the difference? |  |
| Mean of column A | 564.0 |
| Mean of column B | 876.7 |
| Difference between means (B - A) ± SEM | 312.7 ± 71.32 |
| 95% confidence interval | 114.6 to 510.7 |
| R squared (eta squared) | 0.8277 |
|  |  |
| F test to compare variances |  |
| F, DFn, Dfd | 4.341, 2, 2 |
| P value | 0.3745 |
| P value summary | ns |
| Significantly different (P < 0.05)? | No |

**Clone number: HuCCT1**

| PBS | CsEVs |
| --- | --- |
| 308 | 646 |
| 172 | 700 |
| 299 | 483 |
| 282 | 590 |
| 156 | 538 |

| **Test for normal distribution** |  |  |
| --- | --- | --- |
| Shapiro-Wilk test | PBS | CsEVs |
| W | 0.8068 | 0.9871 |
| P value | 0.0920 | 0.9685 |
| Passed normality test (alpha=0.05)? | Yes | Yes |
| P value summary | ns | ns |
|  |  |  |
| Kolmogorov-Smirnov test | PBS | CsEVs |
| KS distance | 0.3008 | 0.1380 |
| P value | >0.1000 | >0.1000 |
| Passed normality test (alpha=0.05)? | Yes | Yes |
| P value summary | ns | ns |

| Column B | CsEVs |
| --- | --- |
| vs. | vs. |
| Column A | PBS |
|  |  |
| Unpaired t test |  |
| P value | 0.0001 |
| P value summary | *** |
| Significantly different (P < 0.05)? | Yes |
| One- or two-tailed P value? | Two-tailed |
| t, df | t=6.900, df=8 |
|  |  |
| How big is the difference? |  |
| Mean of column A | 243.4 |
| Mean of column B | 591.4 |
| Difference between means (B - A) ± SEM | 348.0 ± 50.43 |
| 95% confidence interval | 231.7 to 464.3 |
| R squared (eta squared) | 0.8561 |
|  |  |
| F test to compare variances |  |
| F, DFn, Dfd | 1.367, 4, 4 |
| P value | 0.7693 |
| P value summary | ns |
| Significantly different (P < 0.05)? | No |

**Nucleotides percentage (%)****: RBE**

|  | G0/G1 | | | | S | | | | G2/M | | | |
| --- | --- | --- | --- | --- | --- | --- | --- | --- | --- | --- | --- | --- |
| PBS | 53.63 | 52.83 | 52.74 | 52.14 | 34.42 | 33.51 | 33.33 | 34.76 | 11.95 | 13.65 | 13.93 | 13.11 |
| CsEVs | 42.10 | 41.73 | 42.97 | 42.31 | 40.15 | 40.18 | 40.05 | 39.79 | 17.75 | 18.08 | 16.98 | 17.90 |

**Proliferation index (%): RBE**

| PBS | CsEVs |
| --- | --- |
| 46.37 | 57.9 |
| 47.16 | 58.27 |
| 47.26 | 57.03 |
| 47.87 | 57.69 |

| **Test for normal distribution** |  |  |
| --- | --- | --- |
| Shapiro-Wilk test | PBS | CsEVs |
| W | 0.9642 | 0.9694 |
| P value | 0.8051 | 0.8376 |
| Passed normality test (alpha=0.05)? | Yes | Yes |
| P value summary | ns | ns |

| Column B | CsEVs |
| --- | --- |
| vs. | vs. |
| Column A | PBS |
|  |  |
| Unpaired t test |  |
| P value | <0.0001 |
| P value summary | **** |
| Significantly different (P < 0.05)? | Yes |
| One- or two-tailed P value? | Two-tailed |
| t, df | t=26.19, df=6 |
|  |  |
| How big is the difference? |  |
| Mean of column A | 47.17 |
| Mean of column B | 57.72 |
| Difference between means (B - A) ± SEM | 10.56 ± 0.4031 |
| 95% confidence interval | 9.571 to 11.54 |
| R squared (eta squared) | 0.9913 |
|  |  |
| F test to compare variances |  |
| F, DFn, Dfd | 1.402, 3, 3 |
| P value | 0.7880 |
| P value summary | ns |
| Significantly different (P < 0.05)? | No |

**Nucleotides percentage (%): HuCCT1**

|  | G0/G1 | | | | S | | | | G2/M | | | |
| --- | --- | --- | --- | --- | --- | --- | --- | --- | --- | --- | --- | --- |
| PBS | 61.03 | 60.66 | 61.81 | 60.29 | 25.75 | 26.79 | 26.34 | 27.5 | 13.22 | 12.56 | 11.85 | 12.21 |
| CsEVs | 52.2 | 52.35 | 51.48 | 52.8 | 31.49 | 31.42 | 31.87 | 30.09 | 16.31 | 16.22 | 16.65 | 17.11 |

**Proliferation index (%): HuCCT1**

| PBS | CsEVs |
| --- | --- |
| 38.97 | 47.8 |
| 39.35 | 47.64 |
| 38.19 | 48.52 |
| 39.71 | 47.2 |

| **Test for normal distribution** |  |  |
| --- | --- | --- |
| Shapiro-Wilk test | PBS | CsEVs |
| W | 0.9653 | 0.9650 |
| P value | 0.8120 | 0.8104 |
| Passed normality test (alpha=0.05)? | Yes | Yes |
| P value summary | ns | ns |

| Column B | CsEVs |
| --- | --- |
| vs. | vs. |
| Column A | PBS |
|  |  |
| Unpaired t test |  |
| P value | <0.0001 |
| P value summary | **** |
| Significantly different (P < 0.05)? | Yes |
| One- or two-tailed P value? | Two-tailed |
| t, df | t=20.52, df=6 |
|  |  |
| How big is the difference? |  |
| Mean of column A | 39.06 |
| Mean of column B | 47.79 |
| Difference between means (B - A) ± SEM | 8.735 ± 0.4257 |
| 95% confidence interval | 7.693 to 9.777 |
| R squared (eta squared) | 0.9859 |
|  |  |
| F test to compare variances |  |
| F, DFn, Dfd | 1.407, 3, 3 |
| P value | 0.7857 |
| P value summary | ns |
| Significantly different (P < 0.05)? | No |

**Raw data and statistical analysis of Figure 5:**

**Relative mRNA expression: RBE**

|  | **PBS** | | | **CsEVs** | | |
| --- | --- | --- | --- | --- | --- | --- |
| **cyclin D1** | 0.9449644 | 1.0428155 | 0.9975594 | 1.6227333 | 1.8614855 | 1.7180497 |
| **cyclin D3** | 0.9158608 | 1.0867488 | 0.9971442 | 1.3828846 | 1.3130715 | 1.3372156 |
| **CDK4** | 0.9768523 | 0.9899849 | 1.0258556 | 1.4916123 | 1.4776299 | 1.3981242 |
| **CDK6** | 1.0423447 | 0.9346892 | 1.0129150 | 1.7285950 | 1.8963347 | 1.8389495 |
| **CDK2** | 1.0414523 | 1.0250091 | 0.9272222 | 1.3539810 | 1.4302891 | 1.4000874 |

| **Table Analyzed** | **cyclin D1** |
| --- | --- |
| Column B | CsEVs |
| vs. | vs. |
| Column A | PBS |
|  |  |
| Unpaired t test |  |
| P value | 0.0006 |
| P value summary | *** |
| Significantly different (P < 0.05)? | Yes |
| One- or two-tailed P value? | Two-tailed |
| t, df | t=9.863, df=4 |
|  |  |
| How big is the difference? |  |
| Mean of column A | 0.9951 |
| Mean of column B | 1.734 |
| Difference between means (B - A) ± SEM | 0.7390 ± 0.07493 |
| 95% confidence interval | 0.5309 to 0.9470 |
| R squared (eta squared) | 0.9605 |
|  |  |
| F test to compare variances |  |
| F, DFn, Dfd | 6.023, 2, 2 |
| P value | 0.2848 |
| P value summary | ns |
| Significantly different (P < 0.05)? | No |

| **Table Analyzed** | **cyclin D3** |
| --- | --- |
| Column B | CsEVs |
| vs. | vs. |
| Column A | PBS |
|  |  |
| Unpaired t test |  |
| P value | 0.0030 |
| P value summary | ** |
| Significantly different (P < 0.05)? | Yes |
| One- or two-tailed P value? | Two-tailed |
| t, df | t=6.447, df=4 |
|  |  |
| How big is the difference? |  |
| Mean of column A | 0.9999 |
| Mean of column B | 1.344 |
| Difference between means (B - A) ± SEM | 0.3445 ± 0.05343 |
| 95% confidence interval | 0.1961 to 0.4928 |
| R squared (eta squared) | 0.9122 |
|  |  |
| F test to compare variances |  |
| F, DFn, Dfd | 5.812, 2, 2 |
| P value | 0.2936 |
| P value summary | ns |
| Significantly different (P < 0.05)? | No |

| **Table Analyzed** | **CDK4** |
| --- | --- |
| Column B | CsEVs |
| vs. | vs. |
| Column A | PBS |
|  |  |
| Unpaired t test |  |
| P value | 0.0001 |
| P value summary | *** |
| Significantly different (P < 0.05)? | Yes |
| One- or two-tailed P value? | Two-tailed |
| t, df | t=14.06, df=4 |
|  |  |
| How big is the difference? |  |
| Mean of column A | 0.9976 |
| Mean of column B | 1.456 |
| Difference between means (B - A) ± SEM | 0.4582 ± 0.03259 |
| 95% confidence interval | 0.3677 to 0.5487 |
| R squared (eta squared) | 0.9802 |
|  |  |
| F test to compare variances |  |
| F, DFn, Dfd | 3.952, 2, 2 |
| P value | 0.4039 |
| P value summary | ns |
| Significantly different (P < 0.05)? | No |

| **Table Analyzed** | **CDK6** |
| --- | --- |
| Column B | CsEVs |
| vs. | vs. |
| Column A | PBS |
|  |  |
| Unpaired t test |  |
| P value | 0.0001 |
| P value summary | *** |
| Significantly different (P < 0.05)? | Yes |
| One- or two-tailed P value? | Two-tailed |
| t, df | t=14.03, df=4 |
|  |  |
| How big is the difference? |  |
| Mean of column A | 0.9966 |
| Mean of column B | 1.821 |
| Difference between means (B - A) ± SEM | 0.8246 ± 0.05878 |
| 95% confidence interval | 0.6615 to 0.9878 |
| R squared (eta squared) | 0.9801 |
|  |  |
| F test to compare variances |  |
| F, DFn, Dfd | 2.348, 2, 2 |
| P value | 0.5974 |
| P value summary | ns |
| Significantly different (P < 0.05)? | No |

| **Table Analyzed** | **CDK2** |
| --- | --- |
| Column B | CsEVs |
| vs. | vs. |
| Column A | PBS |
|  |  |
| Unpaired t test |  |
| P value | 0.0007 |
| P value summary | *** |
| Significantly different (P < 0.05)? | Yes |
| One- or two-tailed P value? | Two-tailed |
| t, df | t=9.451, df=4 |
|  |  |
| How big is the difference? |  |
| Mean of column A | 0.9979 |
| Mean of column B | 1.395 |
| Difference between means (B - A) ± SEM | 0.3969 ± 0.04199 |
| 95% confidence interval | 0.2803 to 0.5135 |
| R squared (eta squared) | 0.9571 |
|  |  |
| F test to compare variances |  |
| F, DFn, Dfd | 2.582, 2, 2 |
| P value | 0.5583 |
| P value summary | ns |
| Significantly different (P < 0.05)? | No |

**Relative mRNA expression: HuCCT1**

|  | **PBS** | | | | **CsEVs** | | | |
| --- | --- | --- | --- | --- | --- | --- | --- | --- |
| **cyclin D1** | 0.8402289 | 1.0657943 | 1.0998105 |  | 1.7023549 | 1.8316970 | 1.4691059 | 1.5381254 |
| **cyclin D3** | 1.0576718 | 0.9456779 | 0.9866805 |  | 1.4547365 | 1.5390857 | 1.2550364 |  |
| **CDK4** | 1.0744914 | 0.9368875 | 0.9766302 | 1.0127007 | 1.4749178 | 1.4110429 | 1.3172225 |  |
| **CDK6** | 1.0909454 | 0.8886617 | 0.9850689 | 1.0582598 | 2.6199878 | 2.2770654 | 2.1650353 | 2.4256769 |
| **CDK2** | 1.0515567 | 1.0011689 | 0.9047905 | 1.0519862 | 1.5287358 | 1.2142224 | 1.3680492 |  |

| **Table Analyzed** | **cyclin D1** |
| --- | --- |
| Column B | CsEVs |
| vs. | vs. |
| Column A | PBS |
|  |  |
| Unpaired t test |  |
| P value | 0.0031 |
| P value summary | ** |
| Significantly different (P < 0.05)? | Yes |
| One- or two-tailed P value? | Two-tailed |
| t, df | t=5.354, df=5 |
|  |  |
| How big is the difference? |  |
| Mean of column A | 1.002 |
| Mean of column B | 1.635 |
| Difference between means (B - A) ± SEM | 0.6334 ± 0.1183 |
| 95% confidence interval | 0.3293 to 0.9375 |
| R squared (eta squared) | 0.8515 |
|  |  |
| F test to compare variances |  |
| F, DFn, Dfd | 1.342, 3, 2 |
| P value | 0.9078 |
| P value summary | ns |
| Significantly different (P < 0.05)? | No |

| **Table Analyzed** | **cyclin D3** |
| --- | --- |
| Column B | CsEVs |
| vs. | vs. |
| Column A | PBS |
|  |  |
| Unpaired t test |  |
| P value | 0.0097 |
| P value summary | ** |
| Significantly different (P < 0.05)? | Yes |
| One- or two-tailed P value? | Two-tailed |
| t, df | t=4.644, df=4 |
|  |  |
| How big is the difference? |  |
| Mean of column A | 0.9967 |
| Mean of column B | 1.416 |
| Difference between means (B - A) ± SEM | 0.4196 ± 0.09035 |
| 95% confidence interval | 0.1688 to 0.6705 |
| R squared (eta squared) | 0.8436 |
|  |  |
| F test to compare variances |  |
| F, DFn, Dfd | 6.628, 2, 2 |
| P value | 0.2622 |
| P value summary | ns |
| Significantly different (P < 0.05)? | No |

| **Table Analyzed** | **CDK4** |
| --- | --- |
| Column B | CsEVs |
| vs. | vs. |
| Column A | PBS |
|  |  |
| Unpaired t test |  |
| P value | 0.0006 |
| P value summary | *** |
| Significantly different (P < 0.05)? | Yes |
| One- or two-tailed P value? | Two-tailed |
| t, df | t=7.769, df=5 |
|  |  |
| How big is the difference? |  |
| Mean of column A | 1.000 |
| Mean of column B | 1.401 |
| Difference between means (B - A) ± SEM | 0.4009 ± 0.05160 |
| 95% confidence interval | 0.2682 to 0.5335 |
| R squared (eta squared) | 0.9235 |
|  |  |
| F test to compare variances |  |
| F, DFn, Dfd | 1.843, 2, 3 |
| P value | 0.6010 |
| P value summary | ns |
| Significantly different (P < 0.05)? | No |

| **Table Analyzed** | **CDK6** |
| --- | --- |
| Column B | CsEVs |
| vs. | vs. |
| Column A | PBS |
|  |  |
| Unpaired t test |  |
| P value | <0.0001 |
| P value summary | **** |
| Significantly different (P < 0.05)? | Yes |
| One- or two-tailed P value? | Two-tailed |
| t, df | t=12.63, df=6 |
|  |  |
| How big is the difference? |  |
| Mean of column A | 1.006 |
| Mean of column B | 2.372 |
| Difference between means (B - A) ± SEM | 1.366 ± 0.1082 |
| 95% confidence interval | 1.102 to 1.631 |
| R squared (eta squared) | 0.9638 |
|  |  |
| F test to compare variances |  |
| F, DFn, Dfd | 4.812, 3, 3 |
| P value | 0.2294 |
| P value summary | ns |
| Significantly different (P < 0.05)? | No |

| **Table Analyzed** | **CDK2** |
| --- | --- |
| Column B | CsEVs |
| vs. | vs. |
| Column A | PBS |
|  |  |
| Unpaired t test |  |
| P value | 0.0080 |
| P value summary | ** |
| Significantly different (P < 0.05)? | Yes |
| One- or two-tailed P value? | Two-tailed |
| t, df | t=4.263, df=5 |
|  |  |
| How big is the difference? |  |
| Mean of column A | 1.002 |
| Mean of column B | 1.370 |
| Difference between means (B - A) ± SEM | 0.3680 ± 0.08632 |
| 95% confidence interval | 0.1461 to 0.5899 |
| R squared (eta squared) | 0.7842 |
|  |  |
| F test to compare variances |  |
| F, DFn, Dfd | 5.151, 2, 3 |
| P value | 0.2142 |
| P value summary | ns |
| Significantly different (P < 0.05)? | No |

**Relative protein expression: RBE**

|  | **PBS** | | | **CsEVs** | | |
| --- | --- | --- | --- | --- | --- | --- |
| **cyclin D1** | 1 | 1 | 1 | 1.984468 | 1.594986 | 1.778848 |
| **cyclin D3** | 1 | 1 | 1 | 2.193617 | 2.204785 | 2.020569 |
| **CDK4** |  |  |  |  |  |  |
| **CDK6** | 1 | 1 | 1 | 1.780423 | 1.957151 | 1.541111 |
| **CDK2** | 1 | 1 | 1 | 2.557659 | 2.795495 | 2.270530 |

| **Table Analyzed** | **cyclinD1** |
| --- | --- |
| Column B | CsEVs |
| vs. | vs. |
| Column A | PBS |
|  |  |
| Unpaired t test |  |
| P value | 0.0022 |
| P value summary | ** |
| Significantly different (P < 0.05)? | Yes |
| One- or two-tailed P value? | Two-tailed |
| t, df | t=6.988, df=4 |
|  |  |
| How big is the difference? |  |
| Mean of column A | 1.000 |
| Mean of column B | 1.786 |
| Difference between means (B - A) ± SEM | 0.7861 ± 0.1125 |
| 95% confidence interval | 0.4738 to 1.098 |
| R squared (eta squared) | 0.9243 |
|  |  |
| F test to compare variances |  |
| F, DFn, Dfd | Infinity, 2, 2 |
| P value | <0.0001 |
| P value summary | **** |
| Significantly different (P < 0.05)? | Yes |

| **Table Analyzed** | **cyclinD3** |
| --- | --- |
| Column B | CsEVs |
| vs. | vs. |
| Column A | PBS |
|  |  |
| Unpaired t test |  |
| P value | <0.0001 |
| P value summary | **** |
| Significantly different (P < 0.05)? | Yes |
| One- or two-tailed P value? | Two-tailed |
| t, df | t=19.11, df=4 |
|  |  |
| How big is the difference? |  |
| Mean of column A | 1.000 |
| Mean of column B | 2.140 |
| Difference between means (B - A) ± SEM | 1.140 ± 0.05963 |
| 95% confidence interval | 0.9741 to 1.305 |
| R squared (eta squared) | 0.9892 |
|  |  |
| F test to compare variances |  |
| F, DFn, Dfd | Infinity, 2, 2 |
| P value | <0.0001 |
| P value summary | **** |
| Significantly different (P < 0.05)? | Yes |

| **Table Analyzed** | **CDK6** |
| --- | --- |
| Column B | CsEVs |
| vs. | vs. |
| Column A | PBS |
|  |  |
| Unpaired t test |  |
| P value | 0.0032 |
| P value summary | ** |
| Significantly different (P < 0.05)? | Yes |
| One- or two-tailed P value? | Two-tailed |
| t, df | t=6.301, df=4 |
|  |  |
| How big is the difference? |  |
| Mean of column A | 1.000 |
| Mean of column B | 1.760 |
| Difference between means (B - A) ± SEM | 0.7596 ± 0.1206 |
| 95% confidence interval | 0.4249 to 1.094 |
| R squared (eta squared) | 0.9085 |
|  |  |
| F test to compare variances |  |
| F, DFn, Dfd | Infinity, 2, 2 |
| P value | <0.0001 |
| P value summary | **** |
| Significantly different (P < 0.05)? | Yes |

| **Table Analyzed** | **CDK2** |
| --- | --- |
| Column B | CsEVs |
| vs. | vs. |
| Column A | PBS |
|  |  |
| Unpaired t test |  |
| P value | 0.0005 |
| P value summary | *** |
| Significantly different (P < 0.05)? | Yes |
| One- or two-tailed P value? | Two-tailed |
| t, df | t=10.16, df=4 |
|  |  |
| How big is the difference? |  |
| Mean of column A | 1.000 |
| Mean of column B | 2.541 |
| Difference between means (B - A) ± SEM | 1.541 ± 0.1518 |
| 95% confidence interval | 1.120 to 1.963 |
| R squared (eta squared) | 0.9627 |
|  |  |
| F test to compare variances |  |
| F, DFn, Dfd | Infinity, 2, 2 |
| P value | <0.0001 |
| P value summary | **** |
| Significantly different (P < 0.05)? | Yes |

**Relative protein expression: HuCCT1**

|  | **PBS** | | | **CsEVs** | | |
| --- | --- | --- | --- | --- | --- | --- |
| **cyclin D1** | 1 | 1 | 1 | 1.211196 | 1.361236 | 1.545302 |
| **cyclin D3** | 1 | 1 | 1 | 1.337291 | 1.531186 | 1.465081 |
| **CDK4** |  |  |  |  |  |  |
| **CDK6** | 1 | 1 | 1 | 1.407574 | 1.636347 | 1.666621 |
| **CDK2** | 1 | 1 | 1 | 1.956124 | 2.747763 | 3.390281 |

| **Table Analyzed** | **cyclinD1** |
| --- | --- |
| Column B | CsEVs |
| vs. | vs. |
| Column A | PBS |
|  |  |
| Unpaired t test |  |
| P value | 0.0182 |
| P value summary | * |
| Significantly different (P < 0.05)? | Yes |
| One- or two-tailed P value? | Two-tailed |
| t, df | t=3.856, df=4 |
|  |  |
| How big is the difference? |  |
| Mean of column A | 1.000 |
| Mean of column B | 1.373 |
| Difference between means (B - A) ± SEM | 0.3726 ± 0.09661 |
| 95% confidence interval | 0.1043 to 0.6408 |
| R squared (eta squared) | 0.7880 |
|  |  |
| F test to compare variances |  |
| F, DFn, Dfd | Infinity, 2, 2 |
| P value | <0.0001 |
| P value summary | **** |
| Significantly different (P < 0.05)? | Yes |

| **Table Analyzed** | **cyclinD3** |
| --- | --- |
| Column B | CsEVs |
| vs. | vs. |
| Column A | PBS |
|  |  |
| Unpaired t test |  |
| P value | 0.0014 |
| P value summary | ** |
| Significantly different (P < 0.05)? | Yes |
| One- or two-tailed P value? | Two-tailed |
| t, df | t=7.811, df=4 |
|  |  |
| How big is the difference? |  |
| Mean of column A | 1.000 |
| Mean of column B | 1.445 |
| Difference between means (B - A) ± SEM | 0.4445 ± 0.05691 |
| 95% confidence interval | 0.2865 to 0.6025 |
| R squared (eta squared) | 0.9385 |
|  |  |
| F test to compare variances |  |
| F, DFn, Dfd | Infinity, 2, 2 |
| P value | <0.0001 |
| P value summary | **** |
| Significantly different (P < 0.05)? | Yes |

| **Table Analyzed** | **CDK6** |
| --- | --- |
| Column B | CsEVs |
| vs. | vs. |
| Column A | PBS |
|  |  |
| Unpaired t test |  |
| P value | 0.0022 |
| P value summary | ** |
| Significantly different (P < 0.05)? | Yes |
| One- or two-tailed P value? | Two-tailed |
| t, df | t=6.973, df=4 |
|  |  |
| How big is the difference? |  |
| Mean of column A | 1.000 |
| Mean of column B | 1.570 |
| Difference between means (B - A) ± SEM | 0.5702 ± 0.08177 |
| 95% confidence interval | 0.3431 to 0.7972 |
| R squared (eta squared) | 0.9240 |
|  |  |
| F test to compare variances |  |
| F, DFn, Dfd | Infinity, 2, 2 |
| P value | <0.0001 |
| P value summary | **** |
| Significantly different (P < 0.05)? | Yes |

| **Table Analyzed** | **CDK2** |
| --- | --- |
| Column B | CsEVs |
| vs. | vs. |
| Column A | PBS |
|  |  |
| Unpaired t test |  |
| P value | 0.0149 |
| P value summary | * |
| Significantly different (P < 0.05)? | Yes |
| One- or two-tailed P value? | Two-tailed |
| t, df | t=4.094, df=4 |
|  |  |
| How big is the difference? |  |
| Mean of column A | 1.000 |
| Mean of column B | 2.698 |
| Difference between means (B - A) ± SEM | 1.698 ± 0.4148 |
| 95% confidence interval | 0.5465 to 2.850 |
| R squared (eta squared) | 0.8073 |
|  |  |
| F test to compare variances |  |
| F, DFn, Dfd | Infinity, 2, 2 |
| P value | <0.0001 |
| P value summary | **** |
| Significantly different (P < 0.05)? | Yes |

**Raw data and statistical analysis of Figure 6:**

**Wound healing percentage（%）: RBE**

|  | PBS | | | | | CsEVs | | | | |
| --- | --- | --- | --- | --- | --- | --- | --- | --- | --- | --- |
| 24 h | 6.84 | 6.64 | 7.8 | 4.21 | 5.41 | 15.41 | 13.54 | 16.83 | 12.29 | 18.01 |
| 48 h | 11.86 | 10.16 | 13.94 | 10.01 | 10.88 | 28.96 | 25.13 | 25.45 | 23.43 | 27.39 |

| **Table Analyzed** | **24h** |
| --- | --- |
| Column B | CsEVs |
| vs. | vs. |
| Column A | PBS |
|  |  |
| Unpaired t test |  |
| P value | <0.0001 |
| P value summary | **** |
| Significantly different (P < 0.05)? | Yes |
| One- or two-tailed P value? | Two-tailed |
| t, df | t=7.432, df=8 |
|  |  |
| How big is the difference? |  |
| Mean of column A | 6.180 |
| Mean of column B | 15.22 |
| Difference between means (B - A) ± SEM | 9.036 ± 1.216 |
| 95% confidence interval | 6.232 to 11.84 |
| R squared (eta squared) | 0.8735 |
|  |  |
| F test to compare variances |  |
| F, DFn, Dfd | 2.817, 4, 4 |
| P value | 0.3399 |
| P value summary | ns |
| Significantly different (P < 0.05)? | No |

| **Table Analyzed** | **48h** |
| --- | --- |
| Column B | CsEVs |
| vs. | vs. |
| Column A | PBS |
|  |  |
| Unpaired t test |  |
| P value | <0.0001 |
| P value summary | **** |
| Significantly different (P < 0.05)? | Yes |
| One- or two-tailed P value? | Two-tailed |
| t, df | t=12.27, df=8 |
|  |  |
| How big is the difference? |  |
| Mean of column A | 11.37 |
| Mean of column B | 26.07 |
| Difference between means (B - A) ± SEM | 14.70 ± 1.199 |
| 95% confidence interval | 11.94 to 17.47 |
| R squared (eta squared) | 0.9495 |
|  |  |
| F test to compare variances |  |
| F, DFn, Dfd | 1.763, 4, 4 |
| P value | 0.5963 |
| P value summary | ns |
| Significantly different (P < 0.05)? | No |

**Wound healing percentage（%）: HuCCT1**

|  | PBS | | | | | CsEVs | | | | |
| --- | --- | --- | --- | --- | --- | --- | --- | --- | --- | --- |
| 24 h | 6.52 | 7.35 | 6.35 | 7.59 | 6.25 | 16.62 | 17.25 | 17.03 | 17 | 15.75 |
| 48 h | 12.67 | 12.84 | 10.79 | 13.51 | 10.64 | 24.52 | 24.79 | 26.91 | 24.71 | 28.28 |

| **Table Analyzed** | **24h** |
| --- | --- |
| Column B | CsEVs |
| vs. | vs. |
| Column A | PBS |
|  |  |
| Unpaired t test |  |
| P value | <0.0001 |
| P value summary | **** |
| Significantly different (P < 0.05)? | Yes |
| One- or two-tailed P value? | Two-tailed |
| t, df | t=25.98, df=8 |
|  |  |
| How big is the difference? |  |
| Mean of column A | 6.812 |
| Mean of column B | 16.73 |
| Difference between means (B - A) ± SEM | 9.918 ± 0.3818 |
| 95% confidence interval | 9.038 to 10.80 |
| R squared (eta squared) | 0.9883 |
|  |  |
| F test to compare variances |  |
| F, DFn, Dfd | 1.074, 4, 4 |
| P value | 0.9468 |
| P value summary | ns |
| Significantly different (P < 0.05)? | No |

| **Table Analyzed** | **48h** |
| --- | --- |
| Column B | CsEVs |
| vs. | vs. |
| Column A | PBS |
|  |  |
| Unpaired t test |  |
| P value | <0.0001 |
| P value summary | **** |
| Significantly different (P < 0.05)? | Yes |
| One- or two-tailed P value? | Two-tailed |
| t, df | t=14.52, df=8 |
|  |  |
| How big is the difference? |  |
| Mean of column A | 12.09 |
| Mean of column B | 25.84 |
| Difference between means (B - A) ± SEM | 13.75 ± 0.9468 |
| 95% confidence interval | 11.57 to 15.94 |
| R squared (eta squared) | 0.9635 |
|  |  |
| F test to compare variances |  |
| F, DFn, Dfd | 1.673, 4, 4 |
| P value | 0.6304 |
| P value summary | ns |
| Significantly different (P < 0.05)? | No |

**Migrated cells**

|  | | | | PBS | | | | | | | | | CsEVs | | | | | | | | | |
| --- | --- | --- | --- | --- | --- | --- | --- | --- | --- | --- | --- | --- | --- | --- | --- | --- | --- | --- | --- | --- | --- | --- |
| RBE | 450 | 456 | 460 | | 402 | 417 | 434 | 411 | 411 | 448 | 463 | 688 | | 704 | 686 | 636 | 664 | 678 | 589 | 608 | 631 | 616 |
| HuCCT1 | 437 | 447 | 429 | | 420 | 431 | 387 | 408 | 428 |  |  | 661 | | 610 | 602 | 643 | 595 | 621 | 630 | 569 |  |  |

| **Table Analyzed** | **RBE migration** |
| --- | --- |
| Column B | CsEVs |
| vs. | vs. |
| Column A | PBS |
|  |  |
| Unpaired t test |  |
| P value | <0.0001 |
| P value summary | **** |
| Significantly different (P < 0.05)? | Yes |
| One- or two-tailed P value? | Two-tailed |
| t, df | t=14.92, df=18 |
|  |  |
| How big is the difference? |  |
| Mean of column A | 435.2 |
| Mean of column B | 650.0 |
| Difference between means (B - A) ± SEM | 214.8 ± 14.39 |
| 95% confidence interval | 184.6 to 245.0 |
| R squared (eta squared) | 0.9252 |
|  |  |
| F test to compare variances |  |
| F, DFn, Dfd | 2.876, 9, 9 |
| P value | 0.1314 |
| P value summary | ns |
| Significantly different (P < 0.05)? | No |

| **Table Analyzed** | **HuCCT1 migration** |
| --- | --- |
| Column B | CsEVs |
| vs. | vs. |
| Column A | PBS |
|  |  |
| Unpaired t test |  |
| P value | <0.0001 |
| P value summary | **** |
| Significantly different (P < 0.05)? | Yes |
| One- or two-tailed P value? | Two-tailed |
| t, df | t=15.87, df=14 |
|  |  |
| How big is the difference? |  |
| Mean of column A | 423.4 |
| Mean of column B | 616.4 |
| Difference between means (B - A) ± SEM | 193.0 ± 12.16 |
| 95% confidence interval | 166.9 to 219.1 |
| R squared (eta squared) | 0.9473 |
|  |  |
| F test to compare variances |  |
| F, DFn, Dfd | 2.414, 7, 7 |
| P value | 0.2678 |
| P value summary | ns |
| Significantly different (P < 0.05)? | No |

**Invasive cells**

|  | | | | | PBS | | | | | | | | CsEVs | | | | | |
| --- | --- | --- | --- | --- | --- | --- | --- | --- | --- | --- | --- | --- | --- | --- | --- | --- | --- | --- |
| RBE | 140 | 127 | 143 | 123 | | 126 | 130 | 137 | 128 | 257 | 248 | 277 | | 292 | 278 | 254 | 274 | 280 |
| HuCCT1 | 82 | 72 | 74 | 88 | | 77 | 87 | 77 | 72 | 242 | 218 | 190 | | 246 | 180 | 206 | 185 | 195 |

| **Table Analyzed** | **RBE invasion** |
| --- | --- |
| Column B | CsEVs |
| vs. | vs. |
| Column A | PBS |
|  |  |
| Unpaired t test |  |
| P value | <0.0001 |
| P value summary | **** |
| Significantly different (P < 0.05)? | Yes |
| One- or two-tailed P value? | Two-tailed |
| t, df | t=23.17, df=14 |
|  |  |
| How big is the difference? |  |
| Mean of column A | 131.8 |
| Mean of column B | 270.0 |
| Difference between means (B - A) ± SEM | 138.3 ± 5.966 |
| 95% confidence interval | 125.5 to 151.0 |
| R squared (eta squared) | 0.9746 |
|  |  |
| F test to compare variances |  |
| F, DFn, Dfd | 4.366, 7, 7 |
| P value | 0.0706 |
| P value summary | ns |
| Significantly different (P < 0.05)? | No |

| **Table Analyzed** | **HuCCT1 invasion** |
| --- | --- |
| Column B | CsEVs |
| vs. | vs. |
| Column A | PBS |
|  |  |
| Unpaired t test |  |
| P value | <0.0001 |
| P value summary | **** |
| Significantly different (P < 0.05)? | Yes |
| One- or two-tailed P value? | Two-tailed |
| t, df | t=13.96, df=14 |
|  |  |
| How big is the difference? |  |
| Mean of column A | 78.63 |
| Mean of column B | 207.8 |
| Difference between means (B - A) ± SEM | 129.1 ± 9.252 |
| 95% confidence interval | 109.3 to 149.0 |
| R squared (eta squared) | 0.9329 |
|  |  |
| F test to compare variances |  |
| F, DFn, Dfd | 15.89, 7, 7 |
| P value | 0.0017 |
| P value summary | ** |
| Significantly different (P < 0.05)? | Yes |

**Number of metastatic nodules per lung**

| **RBE Con** | **RBE CsEVs** |
| --- | --- |
| 3 | 5 |
| 1 | 6 |
| 4 | 3 |
| 0 | 8 |
| 0 | 3 |
| 0 | 4 |
| 0 | 2 |
| 0 | 1 |
| 0 | 0 |
| 0 | 0 |

| Column B | **RBE CsEVs** |
| --- | --- |
| vs. | vs. |
| Column A | **RBE Con** |
|  |  |
| Unpaired t test |  |
| P value | 0.0211 |
| P value summary | * |
| Significantly different (P < 0.05)? | Yes |
| One- or two-tailed P value? | Two-tailed |
| t, df | t=2.527, df=18 |
|  |  |
| How big is the difference? |  |
| Mean of column A | 0.8000 |
| Mean of column B | 3.200 |
| Difference between means (B - A) ± SEM | 2.400 ± 0.9499 |
| 95% confidence interval | 0.4044 to 4.396 |
| R squared (eta squared) | 0.2618 |
|  |  |
| F test to compare variances |  |
| F, DFn, Dfd | 3.143, 9, 9 |
| P value | 0.1032 |
| P value summary | ns |
| Significantly different (P < 0.05)? | No |

**Raw data and statistical analysis of Figure 7:**

**Relative protein expression: RBE**

|  | **PBS** | | | **CsEVs** | | |
| --- | --- | --- | --- | --- | --- | --- |
| **E-cadherin** | 1 | 1 | 1 | 0.85789 | 0.48681 | 0.520945 |
| **N-cadherin** | 1 | 1 | 1 | 1.480291 | 1.491019 | 1.360893 |
| **Vimentin** | 1 | 1 | 1 | 1.168817 | 1.236415 | 1.331119 |
| **Slug** | 1 | 1 | 1 | 4.443504 | 3.610421 | 3.579281 |

| **Table Analyzed** | **E-cad** |
| --- | --- |
| Column B | CsEVs |
| vs. | vs. |
| Column A | PBS |
|  |  |
| Unpaired t test |  |
| P value | 0.0331 |
| P value summary | * |
| Significantly different (P < 0.05)? | Yes |
| One- or two-tailed P value? | Two-tailed |
| t, df | t=3.193, df=4 |
|  |  |
| How big is the difference? |  |
| Mean of column A | 1.000 |
| Mean of column B | 0.6219 |
| Difference between means (B - A) ± SEM | -0.3781 ± 0.1184 |
| 95% confidence interval | -0.7069 to -0.04935 |
| R squared (eta squared) | 0.7182 |
|  |  |
| F test to compare variances |  |
| F, DFn, Dfd | Infinity, 2, 2 |
| P value | <0.0001 |
| P value summary | **** |
| Significantly different (P < 0.05)? | Yes |

| **Table Analyzed** | **N-cad** |
| --- | --- |
| Column B | CsEVs |
| vs. | vs. |
| Column A | PBS |
|  |  |
| Unpaired t test |  |
| P value | 0.0004 |
| P value summary | *** |
| Significantly different (P < 0.05)? | Yes |
| One- or two-tailed P value? | Two-tailed |
| t, df | t=10.65, df=4 |
|  |  |
| How big is the difference? |  |
| Mean of column A | 1.000 |
| Mean of column B | 1.444 |
| Difference between means (B - A) ± SEM | 0.4441 ± 0.04170 |
| 95% confidence interval | 0.3283 to 0.5599 |
| R squared (eta squared) | 0.9659 |
|  |  |
| F test to compare variances |  |
| F, DFn, Dfd | Infinity, 2, 2 |
| P value | <0.0001 |
| P value summary | **** |
| Significantly different (P < 0.05)? | Yes |

| **Table Analyzed** | **vimentin** |
| --- | --- |
| Column B | CsEVs |
| vs. | vs. |
| Column A | PBS |
|  |  |
| Unpaired t test |  |
| P value | 0.0065 |
| P value summary | ** |
| Significantly different (P < 0.05)? | Yes |
| One- or two-tailed P value? | Two-tailed |
| t, df | t=5.215, df=4 |
|  |  |
| How big is the difference? |  |
| Mean of column A | 1.000 |
| Mean of column B | 1.245 |
| Difference between means (B - A) ± SEM | 0.2455 ± 0.04707 |
| 95% confidence interval | 0.1148 to 0.3761 |
| R squared (eta squared) | 0.8718 |
|  |  |
| F test to compare variances |  |
| F, DFn, Dfd | Infinity, 2, 2 |
| P value | <0.0001 |
| P value summary | **** |
| Significantly different (P < 0.05)? | Yes |

| **Table Analyzed** | **slug** |
| --- | --- |
| Column B | CsEVs |
| vs. | vs. |
| Column A | PBS |
|  |  |
| Unpaired t test |  |
| P value | 0.0005 |
| P value summary | *** |
| Significantly different (P < 0.05)? | Yes |
| One- or two-tailed P value? | Two-tailed |
| t, df | t=10.17, df=4 |
|  |  |
| How big is the difference? |  |
| Mean of column A | 1.000 |
| Mean of column B | 3.878 |
| Difference between means (B - A) ± SEM | 2.878 ± 0.2830 |
| 95% confidence interval | 2.092 to 3.664 |
| R squared (eta squared) | 0.9627 |
|  |  |
| F test to compare variances |  |
| F, DFn, Dfd | Infinity, 2, 2 |
| P value | <0.0001 |
| P value summary | **** |
| Significantly different (P < 0.05)? | Yes |

**Relative protein expression: HuCCT1**

|  | **PBS** | | | **CsEVs** | | |
| --- | --- | --- | --- | --- | --- | --- |
| **E-cadherin** | 1 | 1 | 1 | 0.644081 | 0.342789 | 0.408535 |
| **N-cadherin** | 1 | 1 | 1 | 2.00475 | 1.548917 | 1.910794 |
| **Vimentin** | 1 | 1 | 1 | 1.658404 | 1.390339 | 1.448152 |
| **Slug** | 1 | 1 | 1 | 2.273115 | 2.742139 | 3.043274 |

| **Table Analyzed** | **E-cad** |
| --- | --- |
| Column B | CsEVs |
| vs. | vs. |
| Column A | PBS |
|  |  |
| Unpaired t test |  |
| P value | 0.0043 |
| P value summary | ** |
| Significantly different (P < 0.05)? | Yes |
| One- or two-tailed P value? | Two-tailed |
| t, df | t=5.848, df=4 |
|  |  |
| How big is the difference? |  |
| Mean of column A | 1.000 |
| Mean of column B | 0.4651 |
| Difference between means (B - A) ± SEM | -0.5349 ± 0.09146 |
| 95% confidence interval | -0.7888 to -0.2809 |
| R squared (eta squared) | 0.8953 |
|  |  |
| F test to compare variances |  |
| F, DFn, Dfd | Infinity, 2, 2 |
| P value | <0.0001 |
| P value summary | **** |
| Significantly different (P < 0.05)? | Yes |

| **Table Analyzed** | **N-cad** |
| --- | --- |
| Column B | CsEVs |
| vs. | vs. |
| Column A | PBS |
|  |  |
| Unpaired t test |  |
| P value | 0.0041 |
| P value summary | ** |
| Significantly different (P < 0.05)? | Yes |
| One- or two-tailed P value? | Two-tailed |
| t, df | t=5.912, df=4 |
|  |  |
| How big is the difference? |  |
| Mean of column A | 1.000 |
| Mean of column B | 1.821 |
| Difference between means (B - A) ± SEM | 0.8215 ± 0.1390 |
| 95% confidence interval | 0.4357 to 1.207 |
| R squared (eta squared) | 0.8973 |
|  |  |
| F test to compare variances |  |
| F, DFn, Dfd | Infinity, 2, 2 |
| P value | <0.0001 |
| P value summary | **** |
| Significantly different (P < 0.05)? | Yes |

| **Table Analyzed** | **vimentin** |
| --- | --- |
| Column B | CsEVs |
| vs. | vs. |
| Column A | PBS |
|  |  |
| Unpaired t test |  |
| P value | 0.0036 |
| P value summary | ** |
| Significantly different (P < 0.05)? | Yes |
| One- or two-tailed P value? | Two-tailed |
| t, df | t=6.126, df=4 |
|  |  |
| How big is the difference? |  |
| Mean of column A | 1.000 |
| Mean of column B | 1.499 |
| Difference between means (B - A) ± SEM | 0.4990 ± 0.08145 |
| 95% confidence interval | 0.2728 to 0.7251 |
| R squared (eta squared) | 0.9037 |
|  |  |
| F test to compare variances |  |
| F, DFn, Dfd | Infinity, 2, 2 |
| P value | <0.0001 |
| P value summary | **** |
| Significantly different (P < 0.05)? | Yes |

| **Table Analyzed** | **slug** |
| --- | --- |
| Column B | CsEVs |
| vs. | vs. |
| Column A | PBS |
|  |  |
| Unpaired t test |  |
| P value | 0.0017 |
| P value summary | ** |
| Significantly different (P < 0.05)? | Yes |
| One- or two-tailed P value? | Two-tailed |
| t, df | t=7.525, df=4 |
|  |  |
| How big is the difference? |  |
| Mean of column A | 1.000 |
| Mean of column B | 2.686 |
| Difference between means (B - A) ± SEM | 1.686 ± 0.2241 |
| 95% confidence interval | 1.064 to 2.308 |
| R squared (eta squared) | 0.9340 |
|  |  |
| F test to compare variances |  |
| F, DFn, Dfd | Infinity, 2, 2 |
| P value | <0.0001 |
| P value summary | **** |
| Significantly different (P < 0.05)? | Yes |

**Relative protein expression: RBE**

|  | **CsEVs+siCon** | | | **CsEVs+siSlug** | | |
| --- | --- | --- | --- | --- | --- | --- |
| **E-cadherin** | 1 | 1 | 1 | 2.323199 | 1.789948 | 1.426422 |
| **N-cadherin** | 1 | 1 | 1 | 0.510679 | 0.80666 | 0.78317 |
| **Vimentin** | 1 | 1 | 1 | 0.394115 | 0.476807 | 0.409036 |
| **Slug** | 1 | 1 | 1 | 0.755325 | 0.783764 | 0.680856 |

| **Table Analyzed** | **E-cad** |
| --- | --- |
| Column B | **CsEVs+siSlug** |
| vs. | vs. |
| Column A | **CsEVs+siCon** |
|  |  |
| Unpaired t test |  |
| P value | 0.0314 |
| P value summary | * |
| Significantly different (P < 0.05)? | Yes |
| One- or two-tailed P value? | Two-tailed |
| t, df | t=3.251, df=4 |
|  |  |
| How big is the difference? |  |
| Mean of column A | 1.000 |
| Mean of column B | 1.847 |
| Difference between means (B - A) ± SEM | 0.8465 ± 0.2604 |
| 95% confidence interval | 0.1235 to 1.570 |
| R squared (eta squared) | 0.7254 |
|  |  |
| F test to compare variances |  |
| F, DFn, Dfd | Infinity, 2, 2 |
| P value | <0.0001 |
| P value summary | **** |
| Significantly different (P < 0.05)? | Yes |

| **Table Analyzed** | **N-cad** |
| --- | --- |
| Column B | **CsEVs+siSlug** |
| vs. | vs. |
| Column A | **CsEVs+siCon** |
|  |  |
| Unpaired t test |  |
| P value | 0.0343 |
| P value summary | * |
| Significantly different (P < 0.05)? | Yes |
| One- or two-tailed P value? | Two-tailed |
| t, df | t=3.157, df=4 |
|  |  |
| How big is the difference? |  |
| Mean of column A | 1.000 |
| Mean of column B | 0.7002 |
| Difference between means (B - A) ± SEM | -0.2998 ± 0.09499 |
| 95% confidence interval | -0.5636 to -0.03610 |
| R squared (eta squared) | 0.7135 |
|  |  |
| F test to compare variances |  |
| F, DFn, Dfd | Infinity, 2, 2 |
| P value | <0.0001 |
| P value summary | **** |
| Significantly different (P < 0.05)? | Yes |

| **Table Analyzed** | **vimentin** |
| --- | --- |
| Column B | **CsEVs+siSlug** |
| vs. | vs. |
| Column A | **CsEVs+siCon** |
|  |  |
| Unpaired t test |  |
| P value | <0.0001 |
| P value summary | **** |
| Significantly different (P < 0.05)? | Yes |
| One- or two-tailed P value? | Two-tailed |
| t, df | t=22.53, df=4 |
|  |  |
| How big is the difference? |  |
| Mean of column A | 1.000 |
| Mean of column B | 0.4267 |
| Difference between means (B - A) ± SEM | -0.5733 ± 0.02544 |
| 95% confidence interval | -0.6440 to -0.5027 |
| R squared (eta squared) | 0.9922 |
|  |  |
| F test to compare variances |  |
| F, DFn, Dfd | Infinity, 2, 2 |
| P value | <0.0001 |
| P value summary | **** |
| Significantly different (P < 0.05)? | Yes |

| **Table Analyzed** | **Slug** |
| --- | --- |
| Column B | **CsEVs+siSlug** |
| vs. | vs. |
| Column A | **CsEVs+siCon** |
|  |  |
| Unpaired t test |  |
| P value | 0.0011 |
| P value summary | ** |
| Significantly different (P < 0.05)? | Yes |
| One- or two-tailed P value? | Two-tailed |
| t, df | t=8.475, df=4 |
|  |  |
| How big is the difference? |  |
| Mean of column A | 1.000 |
| Mean of column B | 0.7400 |
| Difference between means (B - A) ± SEM | -0.2600 ± 0.03068 |
| 95% confidence interval | -0.3452 to -0.1748 |
| R squared (eta squared) | 0.9472 |
|  |  |
| F test to compare variances |  |
| F, DFn, Dfd | Infinity, 2, 2 |
| P value | <0.0001 |
| P value summary | **** |
| Significantly different (P < 0.05)? | Yes |

**Relative protein expression: HuCCT1**

|  | **CsEVs+siCon** | | | **CsEVs+siSlug** | | |
| --- | --- | --- | --- | --- | --- | --- |
| **E-cadherin** | 1 | 1 | 1 | 1.540505 | 1.723469 | 1.61087 |
| **N-cadherin** | 1 | 1 | 1 | 0.465756 | 0.695074 | 0.735248 |
| **Vimentin** | 1 | 1 | 1 | 0.377729 | 0.521527 | 0.396281 |
| **Slug** | 1 | 1 | 1 | 0.627974 | 0.56722 | 0.588702 |

| **Table Analyzed** | **E-cad** |
| --- | --- |
| Column B | **CsEVs+siSlug** |
| vs. | vs. |
| Column A | **CsEVs+siCon** |
|  |  |
| Unpaired t test |  |
| P value | 0.0003 |
| P value summary | *** |
| Significantly different (P < 0.05)? | Yes |
| One- or two-tailed P value? | Two-tailed |
| t, df | t=11.73, df=4 |
|  |  |
| How big is the difference? |  |
| Mean of column A | 1.000 |
| Mean of column B | 1.625 |
| Difference between means (B - A) ± SEM | 0.6249 ± 0.05328 |
| 95% confidence interval | 0.4770 to 0.7729 |
| R squared (eta squared) | 0.9717 |
|  |  |
| F test to compare variances |  |
| F, DFn, Dfd | Infinity, 2, 2 |
| P value | <0.0001 |
| P value summary | **** |
| Significantly different (P < 0.05)? | Yes |

| **Table Analyzed** | **N-cad** |
| --- | --- |
| Column B | **CsEVs+siSlug** |
| vs. | vs. |
| Column A | **CsEVs+siCon** |
|  |  |
| Unpaired t test |  |
| P value | 0.0118 |
| P value summary | * |
| Significantly different (P < 0.05)? | Yes |
| One- or two-tailed P value? | Two-tailed |
| t, df | t=4.384, df=4 |
|  |  |
| How big is the difference? |  |
| Mean of column A | 1.000 |
| Mean of column B | 0.6320 |
| Difference between means (B - A) ± SEM | -0.3680 ± 0.08394 |
| 95% confidence interval | -0.6010 to -0.1349 |
| R squared (eta squared) | 0.8277 |
|  |  |
| F test to compare variances |  |
| F, DFn, Dfd | Infinity, 2, 2 |
| P value | <0.0001 |
| P value summary | **** |
| Significantly different (P < 0.05)? | Yes |

| **Table Analyzed** | **vimentin** |
| --- | --- |
| Column B | **CsEVs+siSlug** |
| vs. | vs. |
| Column A | **CsEVs+siCon** |
|  |  |
| Unpaired t test |  |
| P value | 0.0002 |
| P value summary | *** |
| Significantly different (P < 0.05)? | Yes |
| One- or two-tailed P value? | Two-tailed |
| t, df | t=12.58, df=4 |
|  |  |
| How big is the difference? |  |
| Mean of column A | 1.000 |
| Mean of column B | 0.4318 |
| Difference between means (B - A) ± SEM | -0.5682 ± 0.04516 |
| 95% confidence interval | -0.6935 to -0.4428 |
| R squared (eta squared) | 0.9754 |
|  |  |
| F test to compare variances |  |
| F, DFn, Dfd | Infinity, 2, 2 |
| P value | <0.0001 |
| P value summary | **** |
| Significantly different (P < 0.05)? | Yes |

| **Table Analyzed** | **Slug** |
| --- | --- |
| Column B | **CsEVs+siSlug** |
| vs. | vs. |
| Column A | **CsEVs+siCon** |
|  |  |
| Unpaired t test |  |
| P value | <0.0001 |
| P value summary | **** |
| Significantly different (P < 0.05)? | Yes |
| One- or two-tailed P value? | Two-tailed |
| t, df | t=22.79, df=4 |
|  |  |
| How big is the difference? |  |
| Mean of column A | 1.000 |
| Mean of column B | 0.5946 |
| Difference between means (B - A) ± SEM | -0.4054 ± 0.01779 |
| 95% confidence interval | -0.4548 to -0.3560 |
| R squared (eta squared) | 0.9924 |
|  |  |
| F test to compare variances |  |
| F, DFn, Dfd | Infinity, 2, 2 |
| P value | <0.0001 |
| P value summary | **** |
| Significantly different (P < 0.05)? | Yes |

**Relative Slug expression**

|  | **CsEVs+siCon** | | | | **CsEVs+siSlug** | | | |
| --- | --- | --- | --- | --- | --- | --- | --- | --- |
| RBE | 1.0559925 | 0.9742782 | 1.0740811 | 0.9142118 | 0.3774174 | 0.4004867 | 0.4284037 | 0.3745301 |
| HuCCT1 | 0.9933287 | 0.841132 | 1.1549613 | 1.0228889 | 0.3817834 | 0.4510852 | 0.5481918 | 0.4280627 |

| **Table Analyzed** | **RBE slug** |
| --- | --- |
| Column B | CsEVs+siSlug |
| vs. | vs. |
| Column A | CsEVs+siCon |
|  |  |
| Unpaired t test |  |
| P value | <0.0001 |
| P value summary | **** |
| Significantly different (P < 0.05)? | Yes |
| One- or two-tailed P value? | Two-tailed |
| t, df | t=15.55, df=6 |
|  |  |
| How big is the difference? |  |
| Mean of column A | 1.005 |
| Mean of column B | 0.3952 |
| Difference between means (B - A) ± SEM | -0.6094 ± 0.03919 |
| 95% confidence interval | -0.7053 to -0.5135 |
| R squared (eta squared) | 0.9758 |
|  |  |
| F test to compare variances |  |
| F, DFn, Dfd | 8.836, 3, 3 |
| P value | 0.1066 |
| P value summary | ns |
| Significantly different (P < 0.05)? | No |

| **Table Analyzed** | **HuCCT1 slug** |
| --- | --- |
| Column B | CsEVs+siSlug |
| vs. | vs. |
| Column A | CsEVs+siCon |
|  |  |
| Unpaired t test |  |
| P value | 0.0003 |
| P value summary | *** |
| Significantly different (P < 0.05)? | Yes |
| One- or two-tailed P value? | Two-tailed |
| t, df | t=7.511, df=6 |
|  |  |
| How big is the difference? |  |
| Mean of column A | 1.003 |
| Mean of column B | 0.4523 |
| Difference between means (B - A) ± SEM | -0.5508 ± 0.07334 |
| 95% confidence interval | -0.7302 to -0.3713 |
| R squared (eta squared) | 0.9039 |
|  |  |
| F test to compare variances |  |
| F, DFn, Dfd | 3.374, 3, 3 |
| P value | 0.3446 |
| P value summary | ns |
| Significantly different (P < 0.05)? | No |

**Raw data and statistical analysis of Figure 8:**

**Relative protein expression: RBE**

|  | **Control** | | | **CsEVs** | | |
| --- | --- | --- | --- | --- | --- | --- |
| **p-IKKα/β/IKK(α+β)** | 0.382523 | 0.497877 | 0.377828 | 0.54889947 | 0.65834734 | 0.52688856 |
| **p-IKBα/IKBα** | 0.170022 | 0.343901 | 0.465232 | 0.93699800 | 1.16232100 | 0.96990200 |
| **p-p65/p65** | 0.406920 | 0.345209 | 0.368850 | 0.90314700 | 0.89724700 | 0.93969900 |

| **Table Analyzed** | **p-IKKα/β/IKK(α+β)** |
| --- | --- |
| Column B | CsEVs |
| vs. | vs. |
| Column A | Control |
|  |  |
| Unpaired t test |  |
| P value | 0.0485 |
| P value summary | * |
| Significantly different (P < 0.05)? | Yes |
| One- or two-tailed P value? | Two-tailed |
| t, df | t=2.807, df=4 |
|  |  |
| How big is the difference? |  |
| Mean of column A | 0.4194 |
| Mean of column B | 0.5780 |
| Difference between means (B - A) ± SEM | 0.1586 ± 0.05651 |
| 95% confidence interval | 0.001733 to 0.3155 |
| R squared (eta squared) | 0.6633 |
|  |  |
| F test to compare variances |  |
| F, DFn, Dfd | 1.072, 2, 2 |
| P value | 0.9651 |
| P value summary | ns |
| Significantly different (P < 0.05)? | No |

| **Table Analyzed** | **p-IKBα/IKBα** |
| --- | --- |
| Column B | CsEVs |
| vs. | vs. |
| Column A | Control |
|  |  |
| Unpaired t test |  |
| P value | 0.0033 |
| P value summary | ** |
| Significantly different (P < 0.05)? | Yes |
| One- or two-tailed P value? | Two-tailed |
| t, df | t=6.288, df=4 |
|  |  |
| How big is the difference? |  |
| Mean of column A | 0.3264 |
| Mean of column B | 1.023 |
| Difference between means (B - A) ± SEM | 0.6967 ± 0.1108 |
| 95% confidence interval | 0.3891 to 1.004 |
| R squared (eta squared) | 0.9081 |
|  |  |
| F test to compare variances |  |
| F, DFn, Dfd | 1.486, 2, 2 |
| P value | 0.8044 |
| P value summary | ns |
| Significantly different (P < 0.05)? | No |

| **Table Analyzed** | **p-p65/p65** |
| --- | --- |
| Column B | CsEVs |
| vs. | vs. |
| Column A | Control |
|  |  |
| Unpaired t test |  |
| P value | <0.0001 |
| P value summary | **** |
| Significantly different (P < 0.05)? | Yes |
| One- or two-tailed P value? | Two-tailed |
| t, df | t=24.15, df=4 |
|  |  |
| How big is the difference? |  |
| Mean of column A | 0.3737 |
| Mean of column B | 0.9134 |
| Difference between means (B - A) ± SEM | 0.5397 ± 0.02235 |
| 95% confidence interval | 0.4777 to 0.6018 |
| R squared (eta squared) | 0.9932 |
|  |  |
| F test to compare variances |  |
| F, DFn, Dfd | 1.833, 2, 2 |
| P value | 0.7059 |
| P value summary | ns |
| Significantly different (P < 0.05)? | No |

**Relative protein expression: HuCCT1**

|  | **Control** | | | **CsEVs** | | |
| --- | --- | --- | --- | --- | --- | --- |
| **p-IKKα/β/IKK(α+β)** | 0.509287 | 0.427605 | 0.409419 | 0.67670718 | 0.60507881 | 0.60914277 |
| **p-IKBα/IKBα** | 0.555916 | 0.679959 | 0.669105 | 1.01763400 | 1.05908300 | 1.13291900 |
| **p-p65/p65** | 0.412214 | 0.426736 | 0.393396 | 0.93071500 | 0.97505200 | 0.80988200 |

| **Table Analyzed** | **p-IKKα/β/IKK(α+β）** |
| --- | --- |
| Column B | CsEVs |
| vs. | vs. |
| Column A | Control |
|  |  |
| Unpaired t test |  |
| P value | 0.0092 |
| P value summary | ** |
| Significantly different (P < 0.05)? | Yes |
| One- or two-tailed P value? | Two-tailed |
| t, df | t=4.715, df=4 |
|  |  |
| How big is the difference? |  |
| Mean of column A | 0.4488 |
| Mean of column B | 0.6303 |
| Difference between means (B - A) ± SEM | 0.1815 ± 0.03851 |
| 95% confidence interval | 0.07463 to 0.2884 |
| R squared (eta squared) | 0.8475 |
|  |  |
| F test to compare variances |  |
| F, DFn, Dfd | 1.748, 2, 2 |
| P value | 0.7278 |
| P value summary | ns |
| Significantly different (P < 0.05)? | No |

| **Table Analyzed** | **p-IKBα/IKBα** |
| --- | --- |
| Column B | CsEVs |
| vs. | vs. |
| Column A | Control |
|  |  |
| Unpaired t test |  |
| P value | 0.0011 |
| P value summary | ** |
| Significantly different (P < 0.05)? | Yes |
| One- or two-tailed P value? | Two-tailed |
| t, df | t=8.354, df=4 |
|  |  |
| How big is the difference? |  |
| Mean of column A | 0.6350 |
| Mean of column B | 1.070 |
| Difference between means (B - A) ± SEM | 0.4349 ± 0.05206 |
| 95% confidence interval | 0.2904 to 0.5794 |
| R squared (eta squared) | 0.9458 |
|  |  |
| F test to compare variances |  |
| F, DFn, Dfd | 1.384, 2, 2 |
| P value | 0.8389 |
| P value summary | ns |
| Significantly different (P < 0.05)? | No |

| **Table Analyzed** | **p-p65/p65** |
| --- | --- |
| Column B | CsEVs |
| vs. | vs. |
| Column A | Control |
|  |  |
| Unpaired t test |  |
| P value | 0.0006 |
| P value summary | *** |
| Significantly different (P < 0.05)? | Yes |
| One- or two-tailed P value? | Two-tailed |
| t, df | t=9.832, df=4 |
|  |  |
| How big is the difference? |  |
| Mean of column A | 0.4108 |
| Mean of column B | 0.9052 |
| Difference between means (B - A) ± SEM | 0.4944 ± 0.05029 |
| 95% confidence interval | 0.3548 to 0.6341 |
| R squared (eta squared) | 0.9603 |
|  |  |
| F test to compare variances |  |
| F, DFn, Dfd | 26.15, 2, 2 |
| P value | 0.0737 |
| P value summary | ns |
| Significantly different (P < 0.05)? | No |

**Relative protein level of p-ERK (normalized to ERK)**

|  | **Control** | | | **CsEVs** | | |
| --- | --- | --- | --- | --- | --- | --- |
| **RBE** | 0.450001 | 0.604702 | 0.579758 | 0.743757 | 0.947360 | 1.023716 |
| **HuCCT1** | 0.526890 | 0.594877 | 0.503035 | 0.997632 | 1.141786 | 1.009958 |

| **Table Analyzed** | **RBE** |
| --- | --- |
| Column B | CsEVs |
| vs. | vs. |
| Column A | Control |
|  |  |
| Unpaired t test |  |
| P value | 0.0202 |
| P value summary | * |
| Significantly different (P < 0.05)? | Yes |
| One- or two-tailed P value? | Two-tailed |
| t, df | t=3.738, df=4 |
|  |  |
| How big is the difference? |  |
| Mean of column A | 0.5448 |
| Mean of column B | 0.9049 |
| Difference between means (B - A) ± SEM | 0.3601 ± 0.09634 |
| 95% confidence interval | 0.09265 to 0.6276 |
| R squared (eta squared) | 0.7775 |
|  |  |
| F test to compare variances |  |
| F, DFn, Dfd | 3.036, 2, 2 |
| P value | 0.4955 |
| P value summary | ns |
| Significantly different (P < 0.05)? | No |

| **Table Analyzed** | **HuCCT1** |
| --- | --- |
| Column B | CsEVs |
| vs. | vs. |
| Column A | Control |
|  |  |
| Unpaired t test |  |
| P value | 0.0007 |
| P value summary | *** |
| Significantly different (P < 0.05)? | Yes |
| One- or two-tailed P value? | Two-tailed |
| t, df | t=9.461, df=4 |
|  |  |
| How big is the difference? |  |
| Mean of column A | 0.5416 |
| Mean of column B | 1.050 |
| Difference between means (B - A) ± SEM | 0.5082 ± 0.05372 |
| 95% confidence interval | 0.3591 to 0.6573 |
| R squared (eta squared) | 0.9572 |
|  |  |
| F test to compare variances |  |
| F, DFn, Dfd | 2.812, 2, 2 |
| P value | 0.5247 |
| P value summary | ns |
| Significantly different (P < 0.05)? | No |

**Relative protein expression: RBE**

|  | **Control** | | | **CsEVs** | | | **CsEVs+BAY** | | |
| --- | --- | --- | --- | --- | --- | --- | --- | --- | --- |
| **p-p65/p65** | 0.599691 | 0.636100 | 0.785636 | 1.259851000 | 1.07767500 | 1.301217000 | 0.637894 | 0.852771 | 0.596969 |
| **N-cadherin** | 0.410670 | 0.568668 | 0.724103 | 1.073226928 | 0.92792523 | 0.859655539 | 0.618861 | 0.441702 | 0.384869 |
| **Vimentin** | 0.610693 | 0.664290 | 0.868333 | 0.998353208 | 1.095277324 | 1.092447347 | 0.762295 | 0.879618 | 0.726624 |

| **Table Analyzed** | **p-p65/p65** |  |  |  |  | |
| --- | --- | --- | --- | --- | --- | --- |
| Data sets analyzed | A-C |  |  |  |  | |
| ANOVA summary |  |  |  |  |  | |
| F | 19.60 |  |  |  |  | |
| P value | 0.0023 |  |  |  |  | |
| P value summary | ** |  |  |  |  | |
| Significant diff. among means (P < 0.05)? | Yes |  |  |  |  | |
| R squared | 0.8673 |  |  |  |  | |
|  |  |  |  |  |  | |
| Brown-Forsythe test |  |  |  |  |  | |
| F (DFn, DFd) | 0.04332 (2, 6) |  |  |  |  | |
| P value | 0.9579 |  |  |  |  | |
| P value summary | ns |  |  |  |  | |
| Are SDs significantly different (P < 0.05)? | No |  |  |  |  |  |
|  |  |  |  |  |  | |
| ANOVA table | SS | DF | MS | F (DFn, DFd) | P value | |
| Treatment (between columns) | 0.5584 | 2 | 0.2792 | F (2, 6) = 19.60 | P=0.0023 | |
| Residual (within columns) | 0.08547 | 6 | 0.01425 |  |  | |
| Total | 0.6439 | 8 |  |  |  | |

| Number of families | 1 |  |  |  |  |
| --- | --- | --- | --- | --- | --- |
| Number of comparisons per family | 3 |  |  |  |  |
| Alpha | 0.05 |  |  |  |  |
| Tukey's multiple comparisons test | Mean Diff. | 95.00% CI of diff. | Significant? | Summary | Adjusted P Value |
| Control vs. CsEVs | -0.5391 | -0.8381 to -0.2401 | Yes | ** | 0.0035 |
| Control vs. CsEVs+BAY | -0.02207 | -0.3211 to 0.2769 | No | ns | 0.9723 |
| CsEVs vs. CsEVs+BAY | 0.5170 | 0.2180 to 0.8160 | Yes | ** | 0.0044 |

| **Table Analyzed** | | | **N-cadherin** | |  |  | |  | | |  | |
| --- | --- | --- | --- | --- | --- | --- | --- | --- | --- | --- | --- | --- |
| Data sets analyzed | | | A-C | |  |  | |  | | |  | |
| ANOVA summary | | |  | |  |  | |  | | |  | |
| F | | | 11.07 | |  |  | |  | | |  | |
| P value | | | 0.0097 | |  |  | |  | | |  | |
| P value summary | | | ** | |  |  | |  | | |  | |
| Significant diff. among means (P < 0.05)? | | | Yes | |  |  | |  | | |  | |
| R squared | | | 0.7867 | |  |  | |  | | |  | |
|  | | |  | |  |  | |  | | |  | |
| Brown-Forsythe test | | |  | |  |  | |  | | |  | |
| F (DFn, DFd) | | | 0.1285 (2, 6) | |  |  | |  | | |  | |
| P value | | | 0.8817 | |  |  | |  | | |  | |
| P value summary | | | ns | |  |  | |  | | |  | |
| Are SDs significantly different (P < 0.05)? | | | No | |  |  | |  | | |  | |
|  | | |  | |  |  | |  | | |  | |
| ANOVA table | | | SS | | DF | MS | | F (DFn, DFd) | | | P value | |
| Treatment (between columns) | | | 0.3788 | | 2 | 0.1894 | | F (2, 6) = 11.07 | | | P=0.0097 | |
| Residual (within columns) | | | 0.1027 | | 6 | 0.01712 | |  | | |  | |
| Total | | | 0.4815 | | 8 |  | |  | | |  | |
| Number of families | 1 | |  | | |  | |  |  | |  |  |
| Number of comparisons per family | 3 | |  | | |  | |  |  | |  |  |
| Alpha | 0.05 | |  | | |  | |  |  | |  |  |
| Tukey's multiple comparisons test | Mean Diff. | | 95.00% CI of diff. | | | Significant? | | Summary | Adjusted P Value | |  |  |
| Control vs. CsEVs | -0.3858 | | -0.7136 to -0.05802 | | | Yes | | * | 0.0261 | |  |  |
| Control vs. CsEVs+BAY | 0.08600 | | -0.2418 to 0.4138 | | | No | | ns | 0.7138 | |  |  |
| CsEVs vs. CsEVs+BAY | 0.4718 | | 0.1440 to 0.7996 | | | Yes | | * | 0.0107 | |  |  |

| **Table Analyzed** | **vimentin** |  |  |  |  |
| --- | --- | --- | --- | --- | --- |
| Data sets analyzed | A-C |  |  |  |  |
| ANOVA summary |  |  |  |  |  |
| F | 10.78 |  |  |  |  |
| P value | 0.0103 |  |  |  |  |
| P value summary | * |  |  |  |  |
| Significant diff. among means (P < 0.05)? | Yes |  |  |  |  |
| R squared | 0.7823 |  |  |  |  |
|  |  |  |  |  |  |
| Brown-Forsythe test |  |  |  |  |  |
| F (DFn, DFd) | 0.3765 (2, 6) |  |  |  |  |
| P value | 0.7014 |  |  |  |  |
| P value summary | ns |  |  |  |  |
| Are SDs significantly different (P < 0.05)? | No |  |  |  |  |
|  |  |  |  |  |  |
| ANOVA table | SS | DF | MS | F (DFn, DFd) | P value |
| Treatment (between columns) | 0.2007 | 2 | 0.1004 | F (2, 6) = 10.78 | P=0.0103 |
| Residual (within columns) | 0.05586 | 6 | 0.009310 |  |  |
| Total | 0.2566 | 8 |  |  |  |

| Number of families | 1 |  |  |  |  |
| --- | --- | --- | --- | --- | --- |
| Number of comparisons per family | 3 |  |  |  |  |
| Alpha | 0.05 |  |  |  |  |
| Tukey's multiple comparisons test | Mean Diff. | 95.00% CI of diff. | Significant? | Summary | Adjusted P Value |
| Control vs. CsEVs | -0.3476 | -0.5893 to -0.1059 | Yes | * | 0.0107 |
| Control vs. CsEVs+BAY | -0.07507 | -0.3168 to 0.1667 | No | ns | 0.6300 |
| CsEVs vs. CsEVs+BAY | 0.2725 | 0.03078 to 0.5142 | Yes | * | 0.0312 |

**Relative protein expression: HuCCT1**

|  | **Control** | | | **CsEVs** | | | **CsEVs+BAY** | | | |
| --- | --- | --- | --- | --- | --- | --- | --- | --- | --- | --- |
| **p-p65/p65** | 0.670660 | 0.789536 | 0.819548 | 1.029778000 | 1.189910000 | 1.119000000 | 0.392834 | 0.647561 | 0.725971 |  |
| **N-cadherin** | 0.594072 | 0.445746 | 0.785756 | 0.976658786 | 0.917272255 | 0.975416063 | 0.423480 | 0.629180 | 0.524709 |  |
| **Vimentin** | 0.634096 | 0.284067 | 0.617673 | 1.143835285 | 0.972826226 | 0.893716571 | 0.472582 | 0.598085 | 0.317907 | |

| **Table Analyzed** | **p-p65/p65** |  |  |  |  |
| --- | --- | --- | --- | --- | --- |
| Data sets analyzed | A-C |  |  |  |  |
| ANOVA summary |  |  |  |  |  |
| F | 14.96 |  |  |  |  |
| P value | 0.0047 |  |  |  |  |
| P value summary | ** |  |  |  |  |
| Significant diff. among means (P < 0.05)? | Yes |  |  |  |  |
| R squared | 0.8330 |  |  |  |  |
|  |  |  |  |  |  |
| Brown-Forsythe test |  |  |  |  |  |
| F (DFn, DFd) | 0.4625 (2, 6) |  |  |  |  |
| P value | 0.6504 |  |  |  |  |
| P value summary | ns |  |  |  |  |
| Are SDs significantly different (P < 0.05)? | No |  |  |  |  |
|  |  |  |  |  |  |
| ANOVA table | SS | DF | MS | F (DFn, DFd) | P value |
| Treatment (between columns) | 0.4286 | 2 | 0.2143 | F (2, 6) = 14.96 | P=0.0047 |
| Residual (within columns) | 0.08595 | 6 | 0.01432 |  |  |
| Total | 0.5145 | 8 |  |  |  |

| Number of families | 1 |  |  |  |  |
| --- | --- | --- | --- | --- | --- |
| Number of comparisons per family | 3 |  |  |  |  |
| Alpha | 0.05 |  |  |  |  |
| Tukey's multiple comparisons test | Mean Diff. | 95.00% CI of diff. | Significant? | Summary | Adjusted P Value |
| Control vs. CsEVs | -0.3530 | -0.6528 to -0.05314 | Yes | * | 0.0260 |
| Control vs. CsEVs+BAY | 0.1711 | -0.1287 to 0.4710 | No | ns | 0.2631 |
| CsEVs vs. CsEVs+BAY | 0.5241 | 0.2243 to 0.8239 | Yes | ** | 0.0041 |

| **Table Analyzed** | **N-cadherin** |  |  |  |  |
| --- | --- | --- | --- | --- | --- |
| Data sets analyzed | A-C |  |  |  |  |
| ANOVA summary |  |  |  |  |  |
| F | 11.52 |  |  |  |  |
| P value | 0.0088 |  |  |  |  |
| P value summary | ** |  |  |  |  |
| Significant diff. among means (P < 0.05)? | Yes |  |  |  |  |
| R squared | 0.7934 |  |  |  |  |
|  |  |  |  |  |  |
| Brown-Forsythe test |  |  |  |  |  |
| F (DFn, DFd) | 1.337 (2, 6) |  |  |  |  |
| P value | 0.3310 |  |  |  |  |
| P value summary | ns |  |  |  |  |
| Are SDs significantly different (P < 0.05)? | No |  |  |  |  |
|  |  |  |  |  |  |
| ANOVA table | SS | DF | MS | F (DFn, DFd) | P value |
| Treatment (between columns) | 0.3134 | 2 | 0.1567 | F (2, 6) = 11.52 | P=0.0088 |
| Residual (within columns) | 0.08158 | 6 | 0.01360 |  |  |
| Total | 0.3949 | 8 |  |  |  |

| Number of families | 1 |  |  |  |  |
| --- | --- | --- | --- | --- | --- |
| Number of comparisons per family | 3 |  |  |  |  |
| Alpha | 0.05 |  |  |  |  |
| Tukey's multiple comparisons test | Mean Diff. | 95.00% CI of diff. | Significant? | Summary | Adjusted P Value |
| Control vs. CsEVs | -0.3479 | -0.6400 to -0.05581 | Yes | * | 0.0248 |
| Control vs. CsEVs+BAY | 0.08274 | -0.2094 to 0.3749 | No | ns | 0.6776 |
| CsEVs vs. CsEVs+BAY | 0.4307 | 0.1385 to 0.7228 | Yes | ** | 0.0095 |

| **Table Analyzed** | | | **vimentin** | |  |  | |  | | |  |
| --- | --- | --- | --- | --- | --- | --- | --- | --- | --- | --- | --- |
| Data sets analyzed | | | A-C | |  |  | |  | | |  |
| ANOVA summary | | |  | |  |  | |  | | |  |
| F | | | 10.72 | |  |  | |  | | |  |
| P value | | | 0.0105 | |  |  | |  | | |  |
| P value summary | | | * | |  |  | |  | | |  |
| Significant diff. among means (P < 0.05)? | | | Yes | |  |  | |  | | |  |
| R squared | | | 0.7813 | |  |  | |  | | |  |
|  | | |  | |  |  | |  | | |  |
| Brown-Forsythe test | | |  | |  |  | |  | | |  |
| F (DFn, DFd) | | | 0.05314 (2, 6) | |  |  | |  | | |  |
| P value | | | 0.9487 | |  |  | |  | | |  |
| P value summary | | | ns | |  |  | |  | | |  |
| Are SDs significantly different (P < 0.05)? | | | No | |  |  | |  | | |  |
|  | | |  | |  |  | |  | | |  |
| ANOVA table | | | SS | | DF | MS | | F (DFn, DFd) | | | P value |
| Treatment (between columns) | | | 0.5362 | | 2 | 0.2681 | | F (2, 6) = 10.72 | | | P=0.0105 |
| Residual (within columns) | | | 0.1501 | | 6 | 0.02502 | |  | | |  |
| Total | | | 0.6864 | | 8 |  | |  | | |  |
| Number of families | 1 | |  | | |  | |  |  | | |
| Number of comparisons per family | 3 | |  | | |  | |  |  | | |
| Alpha | 0.05 | |  | | |  | |  |  | | |
| Tukey's multiple comparisons test | Mean Diff. | | 95.00% CI of diff. | | | Significant? | | Summary | Adjusted P Value | | |
| Control vs. CsEVs | -0.4915 | | -0.8878 to -0.09526 | | | Yes | | * | 0.0208 | | |
| Control vs. CsEVs+BAY | 0.04909 | | -0.3472 to 0.4453 | | | No | | ns | 0.9244 | | |
| CsEVs vs. CsEVs+BAY | 0.5406 | | 0.1443 to 0.9369 | | | Yes | | * | 0.0136 | | |

**Raw data and statistical analysis of S1 Fig:**

**Migrated cells**

|  | **CsEVs+siCon** | | | | | | | | | **CsEVs+siSlug** | | | | | | | | | | | |
| --- | --- | --- | --- | --- | --- | --- | --- | --- | --- | --- | --- | --- | --- | --- | --- | --- | --- | --- | --- | --- | --- |
| **RBE** | 607 | 623 | 593 | 655 | 674 | 660 | 622 | 615 | 602 | | 648 | 394 | 400 | 405 | 437 | 456 | 420 | 400 | 424 | 430 | 428 |
| **HuCCT1** | 604 | 613 | 564 | 615 | 605 | 650 |  |  |  | |  | 442 | 481 | 465 | 417 | 430 | 509 |  |  |  |  |

| **Table Analyzed** | **RBE si migration** |
| --- | --- |
| Column B | CsEVs+si-Slug |
| vs. | vs. |
| Column A | CsEVs+si-Con |
|  |  |
| Unpaired t test |  |
| P value | <0.0001 |
| P value summary | **** |
| Significantly different (P < 0.05)? | Yes |
| One- or two-tailed P value? | Two-tailed |
| t, df | t=19.71, df=18 |
|  |  |
| How big is the difference? |  |
| Mean of column A | 629.9 |
| Mean of column B | 419.4 |
| Difference between means (B - A) ± SEM | -210.5 ± 10.68 |
| 95% confidence interval | -232.9 to -188.1 |
| R squared (eta squared) | 0.9557 |
|  |  |
| F test to compare variances |  |
| F, DFn, Dfd | 1.965, 9, 9 |
| P value | 0.3286 |
| P value summary | ns |
| Significantly different (P < 0.05)? | No |

| **Table Analyzed** | **HuCCT1 si migration** |
| --- | --- |
| Column B | CsEVs+si-Slug |
| vs. | vs. |
| Column A | CsEVs+si-Con |
|  |  |
| Unpaired t test |  |
| P value | <0.0001 |
| P value summary | **** |
| Significantly different (P < 0.05)? | Yes |
| One- or two-tailed P value? | Two-tailed |
| t, df | t=8.409, df=10 |
|  |  |
| How big is the difference? |  |
| Mean of column A | 608.5 |
| Mean of column B | 457.3 |
| Difference between means (B - A) ± SEM | -151.2 ± 17.98 |
| 95% confidence interval | -191.2 to -111.1 |
| R squared (eta squared) | 0.8761 |
|  |  |
| F test to compare variances |  |
| F, DFn, Dfd | 1.553, 5, 5 |
| P value | 0.6409 |
| P value summary | ns |
| Significantly different (P < 0.05)? | No |

**Invasive cells**

|  | **CsEVs+siCon** | | | | | | **CsEVs+siSlug** | | | | | |
| --- | --- | --- | --- | --- | --- | --- | --- | --- | --- | --- | --- | --- |
| **RBE** | 215 | 211 | 201 | 238 | 206 |  | 158 | 151 | 135 | 140 | 153 |  |
| **HuCCT1** | 156 | 176 | 171 | 195 | 162 | 176 | 96 | 106 | 98 | 88 | 96 | 79 |

| **Table Analyzed** | **RBE si invasion** |
| --- | --- |
| Column B | CsEVs+si-Slug |
| vs. | vs. |
| Column A | CsEVs+si-Con |
|  |  |
| Unpaired t test |  |
| P value | <0.0001 |
| P value summary | **** |
| Significantly different (P < 0.05)? | Yes |
| One- or two-tailed P value? | Two-tailed |
| t, df | t=8.682, df=8 |
|  |  |
| How big is the difference? |  |
| Mean of column A | 214.2 |
| Mean of column B | 147.4 |
| Difference between means (B - A) ± SEM | -66.80 ± 7.694 |
| 95% confidence interval | -84.54 to -49.06 |
| R squared (eta squared) | 0.9040 |
|  |  |
| F test to compare variances |  |
| F, DFn, Dfd | 2.242, 4, 4 |
| P value | 0.4535 |
| P value summary | ns |
| Significantly different (P < 0.05)? | No |

| **Table Analyzed** | **HuCCT1 si invasion** |
| --- | --- |
| Column B | CsEVs+si-Slug |
| vs. | vs. |
| Column A | CsEVs+si-Con |
|  |  |
| Unpaired t test |  |
| P value | <0.0001 |
| P value summary | **** |
| Significantly different (P < 0.05)? | Yes |
| One- or two-tailed P value? | Two-tailed |
| t, df | t=11.78, df=10 |
|  |  |
| How big is the difference? |  |
| Mean of column A | 172.7 |
| Mean of column B | 93.83 |
| Difference between means (B - A) ± SEM | -78.83 ± 6.694 |
| 95% confidence interval | -93.75 to -63.92 |
| R squared (eta squared) | 0.9328 |
|  |  |
| F test to compare variances |  |
| F, DFn, Dfd | 2.134, 5, 5 |
| P value | 0.4250 |
| P value summary | ns |
| Significantly different (P < 0.05)? | No |

**Raw data and statistical analysis of S2 Fig:**

**EdU positive cells (%): RBE**

| **PBS** | **CsEVs** | **CsEVs+BAY** |
| --- | --- | --- |
| 29.49 | 41.53 | 34.23 |
| 29.41 | 41.49 | 35.29 |
| 28.17 | 43.14 | 31.97 |
| 26.34 | 39.12 | 30.64 |
| 30.72 | 43.53 | 32.17 |

| ANOVA summary |  |  |  |  |  |
| --- | --- | --- | --- | --- | --- |
| F | 70.87 |  |  |  |  |
| P value | <0.0001 |  |  |  |  |
| P value summary | **** |  |  |  |  |
| Significant diff. among means (P < 0.05)? | Yes |  |  |  |  |
| R squared | 0.9219 |  |  |  |  |
|  |  |  |  |  |  |
| Brown-Forsythe test |  |  |  |  |  |
| F (DFn, DFd) | 0.05132 (2, 12) |  |  |  |  |
| P value | 0.9502 |  |  |  |  |
| P value summary | ns |  |  |  |  |
| Are SDs significantly different (P < 0.05)? | No |  |  |  |  |
|  |  |  |  |  |  |
| Bartlett's test |  |  |  |  |  |
| Bartlett's statistic (corrected) | 0.05329 |  |  |  |  |
| P value | 0.9737 |  |  |  |  |
| P value summary | ns |  |  |  |  |
| Are SDs significantly different (P < 0.05)? | No |  |  |  |  |
|  |  |  |  |  |  |
| ANOVA table | SS | DF | MS | F (DFn, DFd) | P value |
| Treatment (between columns) | 438.1 | 2 | 219.0 | F (2, 12) = 70.87 | P<0.0001 |
| Residual (within columns) | 37.09 | 12 | 3.091 |  |  |
| Total | 475.2 | 14 |  |  |  |

| Number of families | 1 |  |  |  |  |
| --- | --- | --- | --- | --- | --- |
| Number of comparisons per family | 3 |  |  |  |  |
| Alpha | 0.05 |  |  |  |  |
|  |  |  |  |  |  |
| Tukey's multiple comparisons test | Mean Diff. | 95.00% CI of diff. | Significant? | Summary | Adjusted P Value |
| PBS vs. CsEVs | -12.94 | -15.90 to -9.970 | Yes | **** | <0.0001 |
| PBS vs. CsEVs+BAY | -4.034 | -7.000 to -1.068 | Yes | ** | 0.0090 |
| CsEVs vs. CsEVs+BAY | 8.902 | 5.936 to 11.87 | Yes | **** | <0.0001 |

**EdU positive cells (%): HuCCT1**

| **PBS** | **CsEVs** | **CsEVs+BAY** |
| --- | --- | --- |
| 34.49 | 44.22 | 37.65 |
| 32.89 | 51.25 | 38.53 |
| 31.28 | 45.62 | 38.85 |
| 35.09 | 44.5 | 32.06 |
| 31.9 | 43.21 | 36.26 |

| ANOVA summary |  |  |  |  |  |
| --- | --- | --- | --- | --- | --- |
| F | 31.10 |  |  |  |  |
| P value | <0.0001 |  |  |  |  |
| P value summary | **** |  |  |  |  |
| Significant diff. among means (P < 0.05)? | Yes |  |  |  |  |
| R squared | 0.8383 |  |  |  |  |
|  |  |  |  |  |  |
| Brown-Forsythe test |  |  |  |  |  |
| F (DFn, DFd) | 0.1254 (2, 12) |  |  |  |  |
| P value | 0.8833 |  |  |  |  |
| P value summary | ns |  |  |  |  |
| Are SDs significantly different (P < 0.05)? | No |  |  |  |  |
|  |  |  |  |  |  |
| Bartlett's test |  |  |  |  |  |
| Bartlett's statistic (corrected) | 1.536 |  |  |  |  |
| P value | 0.4638 |  |  |  |  |
| P value summary | ns |  |  |  |  |
| Are SDs significantly different (P < 0.05)? | No |  |  |  |  |
|  |  |  |  |  |  |
| ANOVA table | SS | DF | MS | F (DFn, DFd) | P value |
| Treatment (between columns) | 424.5 | 2 | 212.2 | F (2, 12) = 31.10 | P<0.0001 |
| Residual (within columns) | 81.90 | 12 | 6.825 |  |  |
| Total | 506.4 | 14 |  |  |  |

| Number of families | 1 |  |  |  |  |
| --- | --- | --- | --- | --- | --- |
| Number of comparisons per family | 3 |  |  |  |  |
| Alpha | 0.05 |  |  |  |  |
|  |  |  |  |  |  |
| Tukey's multiple comparisons test | Mean Diff. | 95.00% CI of diff. | Significant? | Summary | Adjusted P Value |
| **PBS** vs. **CsEVs** | -12.63 | -17.04 to -8.222 | Yes | **** | <0.0001 |
| **PBS** vs. **CsEVs+BAY** | -3.540 | -7.948 to 0.8680 | No | ns | 0.1227 |
| **CsEVs** vs. **CsEVs+BAY** | 9.090 | 4.682 to 13.50 | Yes | *** | 0.0004 |

**Migrated cells: RBE**

| **Control** | **CsEVs** | **CsEVs+BAY** |
| --- | --- | --- |
| 408 | 585 | 320 |
| 362 | 574 | 255 |
| 325 | 572 | 319 |
| 317 | 542 | 245 |
| 348 | 548 | 238 |

| ANOVA summary |  |  |  |  |  |
| --- | --- | --- | --- | --- | --- |
| F | 101.9 |  |  |  |  |
| P value | <0.0001 |  |  |  |  |
| P value summary | **** |  |  |  |  |
| Significant diff. among means (P < 0.05)? | Yes |  |  |  |  |
| R squared | 0.9444 |  |  |  |  |
|  |  |  |  |  |  |
| Brown-Forsythe test |  |  |  |  |  |
| F (DFn, DFd) | 0.7228 (2, 12) |  |  |  |  |
| P value | 0.5054 |  |  |  |  |
| P value summary | ns |  |  |  |  |
| Are SDs significantly different (P < 0.05)? | No |  |  |  |  |
|  |  |  |  |  |  |
| Bartlett's test |  |  |  |  |  |
| Bartlett's statistic (corrected) | 2.170 |  |  |  |  |
| P value | 0.3379 |  |  |  |  |
| P value summary | ns |  |  |  |  |
| Are SDs significantly different (P < 0.05)? | No |  |  |  |  |
|  |  |  |  |  |  |
| ANOVA table | SS | DF | MS | F (DFn, DFd) | P value |
| Treatment (between columns) | 223836 | 2 | 111918 | F (2, 12) = 101.9 | P<0.0001 |
| Residual (within columns) | 13180 | 12 | 1098 |  |  |
| Total | 237016 | 14 |  |  |  |

| Number of families | 1 |  |  |  |  |
| --- | --- | --- | --- | --- | --- |
| Number of comparisons per family | 3 |  |  |  |  |
| Alpha | 0.05 |  |  |  |  |
|  |  |  |  |  |  |
| Tukey's multiple comparisons test | Mean Diff. | 95.00% CI of diff. | Significant? | Summary | Adjusted P Value |
| **Control** vs. **CsEVs** | -212.2 | -268.1 to -156.3 | Yes | **** | <0.0001 |
| **Control** vs. **CsEVs+BAY** | 76.60 | 20.68 to 132.5 | Yes | ** | 0.0086 |
| **CsEVs** vs. **CsEVs+BAY** | 288.8 | 232.9 to 344.7 | Yes | **** | <0.0001 |

**Migrated cells: HuCCT1**

| **Control** | **CsEVs** | **CsEVs+BAY** |
| --- | --- | --- |
| 416 | 584 | 316 |
| 432 | 600 | 380 |
| 428 | 553 | 353 |
| 346 | 560 | 302 |
| 327 | 495 | 326 |

| ANOVA summary |  |  |  |  |  |
| --- | --- | --- | --- | --- | --- |
| F | 40.34 |  |  |  |  |
| P value | <0.0001 |  |  |  |  |
| P value summary | **** |  |  |  |  |
| Significant diff. among means (P < 0.05)? | Yes |  |  |  |  |
| R squared | 0.8705 |  |  |  |  |
|  |  |  |  |  |  |
| Brown-Forsythe test |  |  |  |  |  |
| F (DFn, DFd) | 0.3088 (2, 12) |  |  |  |  |
| P value | 0.7400 |  |  |  |  |
| P value summary | ns |  |  |  |  |
| Are SDs significantly different (P < 0.05)? | No |  |  |  |  |
|  |  |  |  |  |  |
| Bartlett's test |  |  |  |  |  |
| Bartlett's statistic (corrected) | 0.7493 |  |  |  |  |
| P value | 0.6875 |  |  |  |  |
| P value summary | ns |  |  |  |  |
| Are SDs significantly different (P < 0.05)? | No |  |  |  |  |
|  |  |  |  |  |  |
| ANOVA table | SS | DF | MS | F (DFn, DFd) | P value |
| Treatment (between columns) | 135191 | 2 | 67595 | F (2, 12) = 40.34 | P<0.0001 |
| Residual (within columns) | 20105 | 12 | 1675 |  |  |
| Total | 155296 | 14 |  |  |  |

| Number of families | | 1 |  |  |  |  |
| --- | --- | --- | --- | --- | --- | --- |
| Number of comparisons per family | | 3 |  |  |  |  |
| Alpha | | 0.05 |  |  |  |  |
|  | |  |  |  |  |  |
| Tukey's multiple comparisons test | | Mean Diff. | 95.00% CI of diff. | Significant? | Summary | Adjusted P Value |
| **Control** vs. **CsEVs** | -168.6 | | -237.7 to -99.54 | Yes | **** | <0.0001 |
| **Control** vs. **CsEVs+BAY** | | 54.40 | -14.66 to 123.5 | No | ns | 0.1312 |
| **CsEVs** vs. **CsEVs+BAY** | | 223.0 | 153.9 to 292.1 | Yes | **** | <0.0001 |
